# Hyperoxidation of ether-linked phospholipids accelerates neutrophil extracellular trap formation

**DOI:** 10.1038/s41598-017-15668-z

**Published:** 2017-11-22

**Authors:** Satoshi Yotsumoto, Yuito Muroi, Tatsuya Chiba, Rio Ohmura, Maki Yoneyama, Megumi Magarisawa, Kosuke Dodo, Naoki Terayama, Mikiko Sodeoka, Ryohei Aoyagi, Makoto Arita, Satoko Arakawa, Shigeomi Shimizu, Masato Tanaka

**Affiliations:** 1 Laboratory of Immune Regulation, School of Life Sciences, Tokyo University of Pharmacy and Life Sciences, 1432-1 Horinouchi, Hachiouji, Tokyo, 192-0392 Japan; 2 Synthetic Organic Chemistry Laboratory, RIKEN, 2-1 Hirosawa, Wako, Saitama, 351-0198 Japan; 3 Laboratory for Metabolomics, RIKEN Center for Integrative Medical Sciences (IMS) 1-7-22, Suehiro-cho, Tsurumi-ku, Yokohama, Kanagawa 230-0045 Japan; 4 Cellular and Molecular Epigenetics Laboratory, Graduate School of Medical Life Science, Yokohama City University, 1-7-29, Suehiro-cho, Tsurumi, Yokohama, Kanagawa 230-0045 Japan; 5 Division of Physiological Chemistry and Metabolism, Graduate School of Pharmaceutical Sciences, Keio University, 1-5-30, Shibakoen, Minato-ku, Tokyo, 105-0011 Japan; 6 Department of Pathological Cell Biology, Medical Research Institute, Tokyo Medical and Dental University, 1-5-45, Yushima, Bunkyo-ku, Tokyo, 113-8510 Japan

## Abstract

Because neutrophil extracellular trap (NET) formation is involved in the pathology of a wide variety of diseases, NET-regulating compounds are expected to be useful for the therapies of these diseases. In this study, we identified sulfasalazine (SSZ) as a potent enhancer of NET formation both *in vitro* and *in vivo*. Although SSZ did not increase the amount of ROS generated, it accelerated the generation of ether-linked oxidized phospholipids, such as PE (18;1e/15-HETE) and PC (16;0e/13-HODE). Trolox, but not 2-ME, effectively suppressed lipid oxidation and NET formation that were induced by SSZ. SSZ is known as a potent inducer of ferroptosis in cancer cells by inhibiting xCT, a component of the cystine transporter. However, we found that SSZ accelerated NET formation in an xCT-independent manner. Structure-activity relationship studies revealed that the sulfapyridine moiety of SSZ plays a central role in enhancing NET formation. Furthermore, we found that two additional sulfonamide and sulfone derivatives possess NET-inducing activity by accelerating lipid oxidation. These results indicate that the hyperoxidation of ether-linked phospholipids is a key mechanism for accelerating NET formation.

## Introduction

Polymorphonuclear leukocytes, or neutrophils, are sentinel cells that are part of the first line of defense against bacterial infection. These cells contribute to eliminating invading bacteria via cell killing and phagocytic activity. Recently, another mechanism has been identified as a novel defense mechanism used by neutrophils^1,2^. Activated neutrophils undergo a distinct type of cell death called NETosis that is morphologically distinct from both apoptosis and necrosis^3^. This cell death process has been associated with the extracellular release of chromosomal DNA that is coated with histones and proteases and that forms web-like structures^4^. These structures are named neutrophil extracellular traps (NETs), and they play critical roles in the efficient elimination of bacteria by immobilizing bacterial cells. Although NET formation is generally considered to be a consequence of the NETosis cell death process, NET formation was recently found to occur without cellular suicide^5,6^.

NETs are generated in response to a variety of stimuli, including bacterial components, monosodium urate, cholesterol crystals, and pharmacological reagents, such as phorbol-12-myristate-13 acetate (PMA) and ionomycin. In response to these stimuli, reactive oxygen species (ROS) are generated in an NADPH oxidase-dependent manner. The resulting oxidative stress can induce the activation of protein-arginine deiminase 4 (PAD4)^7,8^. PAD4 is specifically expressed by neutrophils, and activated PAD4 mediates the citrullination of histone H3 in N-terminal arginine residues, which results in the decondensation of DNA and NET formation^7,9,10^. Inhibiting PAD4 activity using chemical inhibitors suppressed NET formation in mouse and human neutrophils^11,12^. PAD4-deficient mice exhibited impaired NET formation and susceptibility to bacterial infection, demonstrating that PAD4 is important during NET formation^8^. However, little is currently known about the signaling pathways that lie downstream of the ROS for PAD4 activation. It has also been reported that neutrophil elastase (NE) is implicated in efficient NET formation. NE is translocated from granules into the nucleus in an ROS- and myeloperoxidase-dependent manner. Once in the nucleus, NE cleaves histones to promote chromatin decondensation^13,14^. In addition to the chromatin decondensation induced by PAD4 and NE, the nuclear membrane must also be disrupted for NET formation to occur. However, the precise mechanisms underlying each of these events remain obscure.

NET formation has recently attracted a great deal of attention not only as a defense mechanism but also because it is implicated in the pathologies underlying a wide variety of diseases^1,15^. During NETosis, dying neutrophils may release intracellular materials, including damage-associated molecular patterns (DAMPs), which can cause the deterioration of tissue injuries or delay regeneration^1^. For instance, neutrophils obtained from diabetic humans and mice are more susceptible to NETosis and, as a consequence, wound healing is delayed in diabetic mice^16^. In individuals with ischemic reperfusion injury, NET formation is mediated by TLR signaling and exacerbates sterile injury^17^. On the other hand, there are some diseases in which NET formation has beneficial effects on pathology. For example, neutrophils undergo NETosis, leading to NET formation at inflamed sites in gout, and the formation of NET leads to the resolution of inflammation by degrading inflammatory cytokines^18^. Furthermore, neutrophils detect human immunodeficiency virus (HIV)-1 by Toll-like receptors (TLRs) 7 and 8, which recognize viral nucleic acids. Engagement of TLRs 7 and 8 induces NET formation, leading to NET-dependent HIV-1 elimination^19^. Because NET formation can have a positive or negative effect on a variety of diseases, NET-regulating compounds are expected to be useful in applications developed as therapies for these diseases.

Here, we show that sulfasalazine (SSZ), a drug that is commonly used to treat inflammatory bowel disease or rheumatoid arthritis, significantly promoted PMA-induced NETosis and the subsequent formation of NETs in isolated mouse and human neutrophils. Consistent with these findings, SSZ decreased the number of activated neutrophils under inflammatory conditions by inducing NETosis *in vivo*. The promotion of NET formation is attributable to the non-enzymatic oxidation of ether-linked phospholipids, indicating that oxidized lipids play a role in NET formation. We also found that two additional sulfonamide and sulfone derivatives that are structurally related to SSZ possess NET-inducing activity by accelerating lipid oxidation. These results indicate that hyperoxidation of phospholipids could be a potential strategy for the enhancing NET formation.

## Results

### Sulfasalazine accelerates cell death in activated neutrophils via NETosis

**Figure 1:**
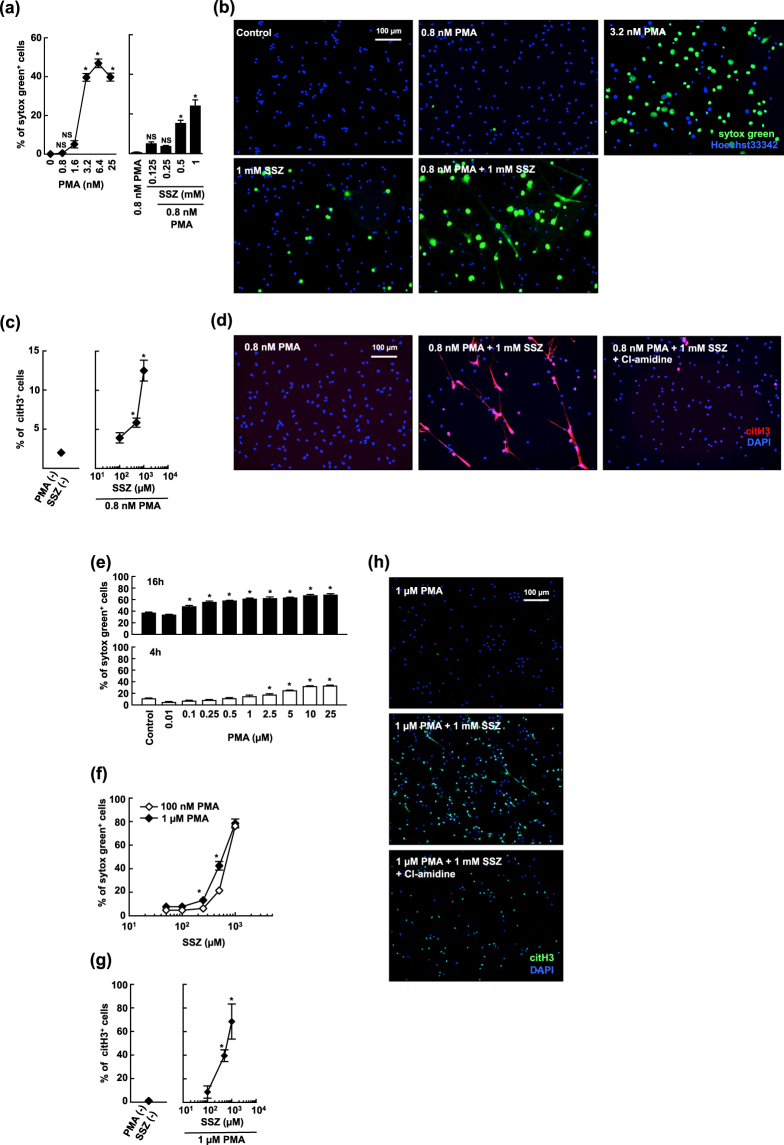
SSZ accelerates cell death in activated neutrophils via NETosis. (**a**) Human peripheral blood neutrophils were stimulated with various concentrations of PMA or/and SSZ for 2.5 h. Cells were stained with sytox green. The proportion of dead cells was determined by counting the number of sytox green^+^ cells using Image-J software. Average values and the s.d. of triplicated samples in a single experiment are shown. *P < 0.01, NS, not significant, one-way ANOVA, compared with PMA (−) (left) or 0.8 nM PMA (right). The data shown are representative of two independent experiments. (**b**) Morphological analysis of SSZ-treated human activated neutrophils. Human neutrophils were stimulated with 3.2 nM PMA alone, 0.8 nM PMA or/and 1 mM SSZ for 2.5 h. Cells were stained using sytox green and Hoechest 33342 and visualized using fluorescence microscopy. (**c**,**d**) Human neutrophils were stimulated with 0.8 nM PMA and various concentrations of SSZ in the presence or absence of 200 µM Cl-amidine for 2.5 h. NET formation was visualized by staining the cells for DNA (DAPI) and with anti-citH3 monoclonal antibody (clone 11-11B-4F). (**b**,**d**) Original magnification, ×20. The data shown are representative of two independent experiments. (**e**,**f**) Mouse neutrophils were stimulated with various concentrations of PMA alone (**e**) for 4 or 16 h, and SSZ with 100 n M or 1 μM PMA (**f**) for 4 h. Cells were stained with sytox green. The proportion of dead cells was determined by counting the number of sytox green^+^ cells using Operetta CLS. *P < 0.01, one-way ANOVA, compared with each control (**e**) or 100 nM PMA (**f**). Average values and the s.d. of triplicated samples in a single experiment are shown. The data shown are representative of two independent experiments. (**g**,**h**) Increased citrullination of histone H3 in SSZ-treated activated neutrophils. Mouse neutrophils were stimulated with 1 µM PMA and various concentrations of SSZ in the presence or absence of 200 µM Cl-amidine for 4 h. Cells were fixed and stained for DNA (DAPI) and with anti-citH3 polyclonal antibody (Abcam). (**c**,**g**) The proportion of cells undergoing NET formation was determined by counting the number of citH3^+^ cells using Image-J software. Average values and the s.d. of triplicated samples in a single experiment are shown. *P < 0.01, one-way ANOVA, compared with PMA (−) and SSZ (−). The data shown are representative of two independent experiments. (**h**) NET formation was visualized by fluorescence microscopy. Original magnification, ×20. The data shown are representative of two independent experiments.

We screened several cell death related compounds for their ability to enhance NETosis and NET formation in activated human peripheral blood neutrophils, and found that SSZ, which is widely used to treat autoimmune diseases, accelerated cell death in activated human neutrophils. PMA at the dose of 3.2 nM was capable of inducing NETosis in human neutrophils, whereas NETosis was not observed when a lower dose of PMA (0.8 nM) was administered (Fig. 1a, left, b). As shown in Fig. 1a, right, b, SSZ enhanced cell death in neutrophils pretreated with 0.8 nM PMA in a dose-dependent manner, although SSZ alone did not induce neutrophil cell death. NETosis has been associated with the citrullination of histone H3. An immunohistochemical analysis performed using anti-citrullinated histone H3 antibodies revealed that treatment with SSZ and PMA increased the number of dead neutrophils that underwent histone H3 citrullination, whereas treatment with 0.8 nM PMA alone did not induce citrullination (Fig. 1c,d). Citrullination was inhibited by adding Cl-amidine, a PAD inhibitor, which confirmed that the observed cell death resulted from NETosis (Fig. 1d). NET formation is induced by not only PMA and ionomycin, but also bacteria, such as *E. coli* ^4,20,21^. Therefore, we next investigated whether SSZ enhanced NET formation by *E. coli*. We found that SSZ enhanced *E. coli*-induced NET formation (Supplemental Fig. 1a).

We also investigated the effects of SSZ on activated mouse neutrophils. It is reported that mouse bone marrow neutrophils undergo NETosis more slowly and less efficiently than human neutrophils^22–25^. Consistent with previous reports, mouse neutrophils required a longer time (16 h) to undergo NETosis even when treated with a high dose of PMA, but hardly died by treatment with up to 1 µM PMA for 4 h (Fig. 1e). However, we found that SSZ rapidly and dose-dependently accelerated cell death in mouse bone marrow neutrophils by stimulation with either 100 nM or 1 µM PMA for 4 h (Fig. 1f). It has been reported that SSZ alone induced apoptosis in neutrophils^26^. However, we found that mouse bone marrow neutrophils treated with 1 mM SSZ for 4 h failed to undergo cell death (Supplemental Fig. 2a,b). Treatment with SSZ and PMA increased the number of dead neutrophils that underwent histone H3 citrullination (Fig. 1g,h). This citrullination was inhibited by adding Cl-amidine, which confirmed that the observed cell death resulted from NETosis (Fig. 1h). We further analyzed the morphological features of dying neutrophils by transmission electron microscopy. Neutrophils treated with PMA alone showed lobulated nuclei with clearly condensed heterochromatin that was distributed in the periphery of nuclei (Supplemental Fig. 3). On the other hand, when treated with PMA and SSZ for 1 h, decondensed chromatin and loss of nuclear envelope were observed in neutrophils (Supplemental Fig. 3, red arrow). Moreover, some dying neutrophils released cytoplasmic contents to form prominent extracellular structures 2 h after stimulation (Supplemental Fig. 3, blue arrows). These findings are consistent with those of previous reports that describe the morphological aspects of NETosis and NETs. We further evaluated the effects of SSZ on neutrophils that were activated using ionomycin, another inducer of NETosis. Immunohistochemical analysis with anti-citrullinated histone H3 antibodies clearly showed that ionomycin induced NETosis in a dose-dependent manner (Supplemental Fig. 4a, left, b), and that SSZ enhanced NETosis in neutrophils that were incubated in the presence of ionomycin (5 μM) (Supplemental Fig. 4a, right, b).

Taken together, these results indicate that SSZ enhances NETosis in mouse and human activated neutrophils.

### SSZ induces neutrophil death via NETosis *in vivo*

**Figure 2:**
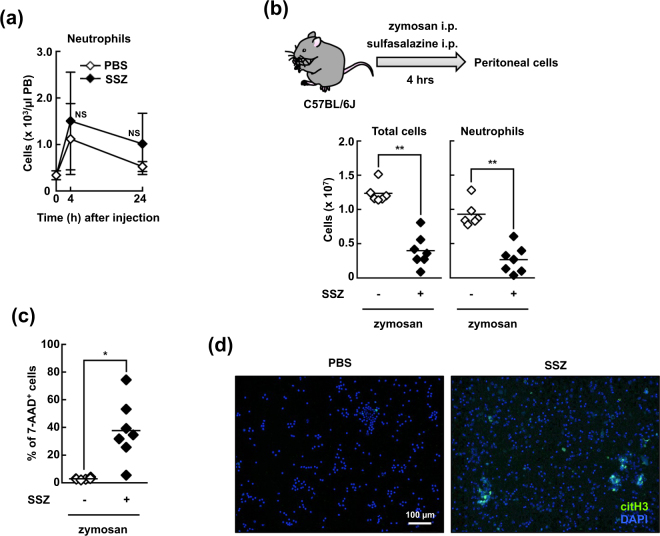
SSZ induces neutrophil death via NETosis *in vivo*. (**a**) WT mice were intraperitoneally injected with 16 mg SSZ or PBS. After 4 or 24 h, the absolute number of CD45.2^+^Ly-6C^−^Ly-6G^+^ neutrophils was determined by counting cells on a microscope using a hemocytometer and analyzing the data using flow cytometry. The average and s.d. of 3 mice are shown. The mean of the number of cells in PBS- and SSZ-injected mice at different time points were compared. NS, not significant between two groups, two-way ANOVA. The data shown are representative of two independent experiments. (**b**–**d**) WT mouse were intraperitoneally injected with 1 mg zymosan and 16 mg SSZ or PBS. After 4 h, peritoneal cells were collected. (**b**) The numbers of total cells (left) and CD45.2^+^Ly-6C^−^Ly-6G^+^ neutrophils (right) were counted as described in (**a**). (**c**) Peritoneal cells were stained with 7-AAD, and the proportion of dead cells (7-AAD^+^ cells) was determined using flow cytometry. The values shown are averages and the s.d. of 6 (SSZ (−)) to 7 (SSZ (+)) mice per group (**b**,**c**). *P < 0.005, **P < 0.001, Student’s t-test. (**d**) Peritoneal cells were visualized with anti-citH3 polyclonal antibody as described. Original magnification, ×20. The data shown are representative of three independent experiments.

We next sought to determine the effect of SSZ on neutrophil death *in vivo*. When SSZ was injected i.p. into naïve mice, the number of neutrophils in the blood was not decreased, suggesting that SSZ exerts no cytocidal effects on neutrophils under physiological conditions (Fig. 2a). We next sought to determine the effect of SSZ on activated neutrophils. The injection of zymosan into the peritoneal cavity induces recruitment of activated neutrophils into the peritoneal cavity^27–30^. Mice were injected intraperitoneally with zymosan and then immediately injected with or without SSZ. As shown in Fig. 2b, a substantial number of immune cells, primarily neutrophils, had accumulated in the peritoneal cavity at 4 h after the injection of zymosan alone, and the number of neutrophils was dramatically reduced by co-injecting the mice with SSZ. Moreover, the proportion of 7-AAD-positive dead cells in all infiltrating cells was dramatically higher in mice treated with both zymosan and SSZ (Fig. 2c). Some of the peritoneal neutrophils in mice injected with zymosan and SSZ exhibited immunoreactivity for anti-citrullinated histone H3 antibodies, indicating that neutrophils undergo NETosis *in vivo* (Fig. 2d). These data suggest that SSZ causes neutrophils to undergo cell death and enhanced NET formation *in vivo*.

### SSZ does not enhance ROS production in activated neutrophils

**Figure 3:**
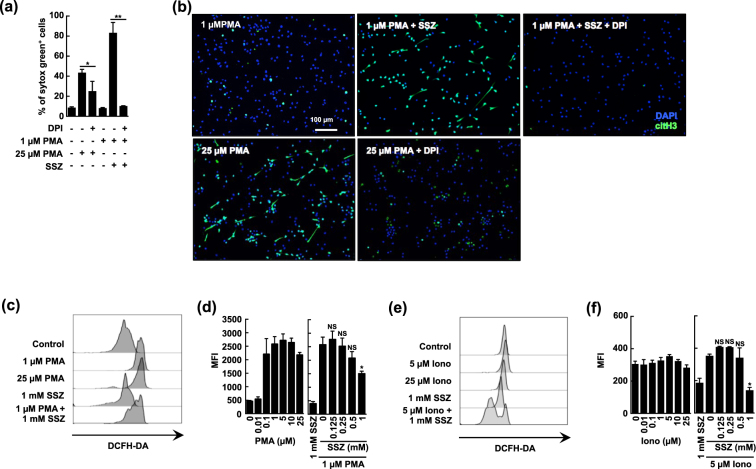
SSZ does not enhance ROS production in activated neutrophils. (**a**,**b**) The effect of the NADPH-oxidase inhibitor, DPI on NET formation. Mouse neutrophils were stimulated with PMA in the presence or absence of 1 mM SSZ and 40 µM DPI for 4 h. (**a**) The proportion of cells undergoing NET formation was determined using sytox green. Average values and the s.d. of triplicated samples in a single experiment are shown. *P < 0.05, **P < 0.01, one-way ANOVA. The data shown are representative of two independent experiments. (**b**) NET formation was visualized by staining the cells for DNA (DAPI) and with anti-citH3 polyclonal antibody. Original magnification, ×20. The data shown are representative of three independent experiments. (**c**–**f**) The effect of SSZ on ROS production. Mouse neutrophils were pre-incubated with an intracellular ROS-indicator, DCFH-DA for 5 min, and then stimulated with various concentrations of PMA and/or SSZ (**c**,**d**), or ionomycin and/or SSZ (**e**,**f**) for 15 min. (**c**,**e**) ROS production was analyzed by flow cytometry. (**d**,**f**) ROS generation was quantified using mean fluorescence intensity (MFI). Average values and the s.d. of triplicated samples in a single experiment are shown. *P < 0.01, one-way ANOVA, compared with 1 µM PMA (**d**) or 5 µM ionomycin (**f**). The data shown are representative of three independent experiments.

Reactive oxygen species (ROS) production is required for PMA-induced NETosis^31^. To explore the mechanisms by which NETosis is accelerated by SSZ, we explored whether SSZ affects ROS production in neutrophils. We first evaluated the effects of diphenyleneiodonium (DPI), a NADPH oxidase inhibitor, on NETosis. As shown in Fig. 3a and b, DPI inhibited NETosis that was induced by either a high dose of PMA or a combination of low-dose PMA and SSZ. These results indicated that the observed SSZ-induced acceleration of NETosis requires NADPH oxidase-induced ROS production. This result suggests that SSZ may enhance the production of ROS in neutrophils. We next measured the amount of ROS produced by using DCFH-DA. PMA stimulated ROS production in a dose-dependent manner (Fig. 3c,d). However, contrary to our expectations, SSZ did not enhance ROS production in neutrophils treated with a low dose of PMA, and 1 mM SSZ rather reduced ROS production in activated neutrophils (Fig. 3c,d). These results indicate that the SSZ-induced acceleration of NETosis requires NADPH oxidase activity, and also that SSZ does not increase the amount of ROS produced in activated neutrophils. We also evaluated the effect of SSZ on ROS production in ionomycin-treated neutrophils and found that SSZ did not enhance, but rather reduced the production of ROS under these conditions (Fig. 3e,f). This may reflect the ROS scavenging effect of SSZ as previously reported^32,33^.

### Accelerated lipid oxidation is essential for SSZ-induced NETosis

**Figure 4:**
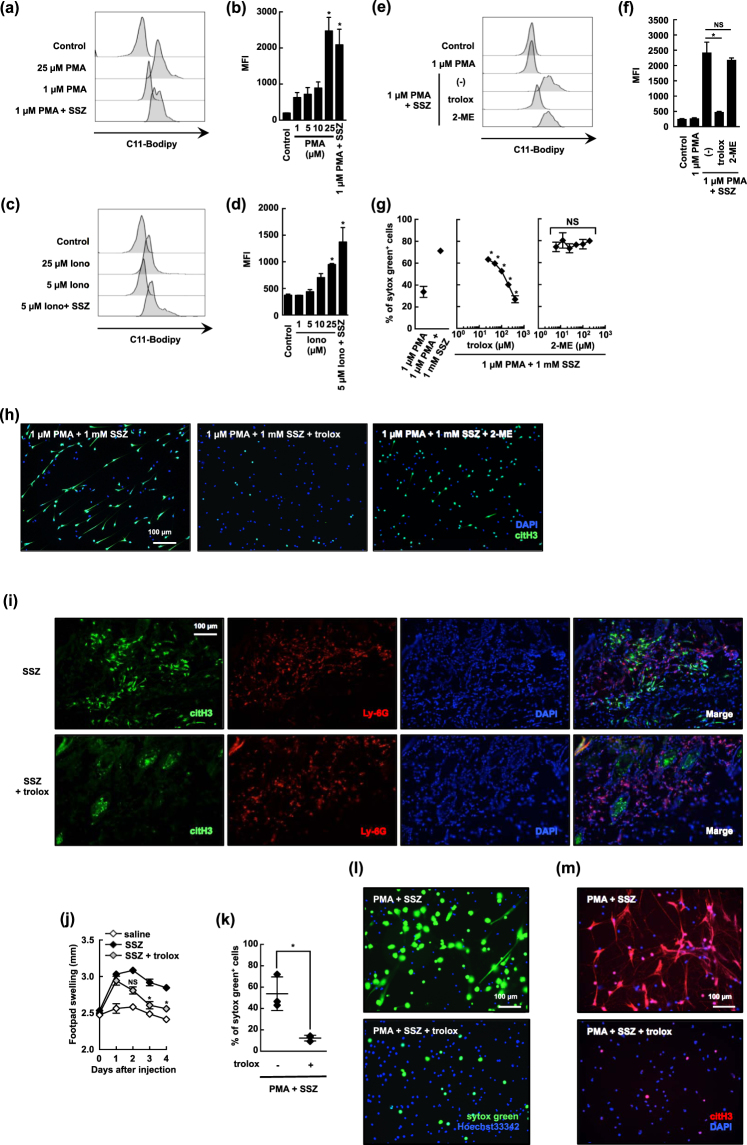
Accelerated lipid oxidation is essential for SSZ-induced NETosis. (**a**–**d**) Mouse neutrophils were stimulated with various concentrations of PMA (**a**,**b**) or ionomycin (**c**,**d**) in the presence or absence of 1 mM SSZ for 1 h. C11-Bodipy^581/591^ was then added. (**a**,**c**) The accumulation of lipid oxidation was analyzed using flow cytometry. (**b**,**d**) Average mean fluorescent intensity (MFI) of C11-Bodipy analysis with s.d. of triplicated samples are shown. *P < 0.01, one-way ANOVA, compared with 1 µM PMA (**b**) or 5 µM ionomycin (**d**). The data shown are representative of three independent experiments. (**e**,**f**) Mouse neutrophils were stimulated with 1 µM PMA alone, 1 µM PMA + 1 mM SSZ in the presence or absence of an antioxidant reagent (400 µM trolox or 200 µM 2-ME) for 1 h. The accumulation of lipid oxidation was determined as described above. (**f**) Average mean fluorescent intensity (MFI) with s.d. of triplicated samples are shown. *P < 0.01, NS, not significant, one-way ANOVA. The data shown are representative of three independent experiments. (**g**,**h**) The effect of antioxidant reagents on SSZ-induced NET formation. Mouse neutrophils were stimulated with 1 µM PMA alone, 1 µM PMA + 1 mM SSZ in the presence or absence of various concentration of trolox and 2-ME. (**g**) Cells were stained with sytox green. The proportion of dead cells was determined by counting the number of sytox green^+^ cells using IN Cell Analyzer 2000. Average values and the s.d. of triplicated samples in a single experiment are shown. *P < 0.01, NS, not significant, one-way ANOVA, compared with 1 µM PMA + 1 mM SSZ. The data shown are representative of three independent experiments. (**h**) NET formation was visualized by staining with DAPI and anti-citH3 antibody. Original magnification, ×20. The data shown are representative of three independent experiments. (**i**,**j**) Trolox inhibit SSZ-induced citrullination of histone H3 in footpad. WT mice were injected with SSZ alone, or SSZ + trolox into footpads. (**i**) After 48 h, the footpads were resected and stained with Ly-6G Ab, DAPI, and anti-citH3 polyclonal antibody (Abcam). Original magnification, ×20. (**j**) Footpad swelling was measured at indicated time. The average and s.d. of 3 mice are shown. *P < 0.001, NS, not significant, one-way ANOVA, compared with SSZ-injected mice. (**k**–**m**) Human neutrophils were stimulated with 0.8 nM PMA + 1 mM SSZ with or without 400 µM trolox for 2.5 h. (**k**) Cells were stained with sytox green and Hoechest 33342. The proportion of dead cells was counted by determining the number of sytox green^+^ cells using Image J software. Average values and the s.d. of triplicated samples in a single experiment are shown. *P < 0.05, Student’s t-test. (**l**) NET formulation was visualized by staining with sytox green and hoechest33342. (**m**) NET formation was visualized by staining with DAPI and anti-citH3 antibody (clone 11-11B-4F). (**l**,**m**) Original magnification, ×20. The data shown are representative of three independent experiments.

We next evaluated the effects of SSZ on ROS-associated responses in activated neutrophils. We found that SSZ accelerated lipid oxidation in activated neutrophils. Lipid oxidation was measured using C11-Bodipy 581/591 dye, which exhibits a shift in fluorescence from red to green when oxidized. As shown in Fig. 4a and b, PMA increased lipid oxidation in a dose-dependent manner. A low dose (1 µM) of PMA induced a slight increase in oxidized C11-Bodipy, and SSZ dramatically accelerated lipid oxidation in neutrophils that were pretreated with a low dose of PMA (Fig. 4a,b). We also found that SSZ increased lipid oxidation in activated neutrophils treated with a low dose (5 µM) of ionomycin (Fig. 4c,d). This lipid hyperoxidation by SSZ was not inhibited by 2-ME but was inhibited by trolox, an analog of vitamin E that functions as an anti-oxidant during lipid oxidation (Fig. 4e,f). Consistent with these results, trolox, but not 2-ME, efficiently and dose-dependently suppressed low-dose PMA and SSZ-induced NETosis (Fig. 4g,h). Observation under transmission electron microscopy also demonstrated that trolox inhibited morphological changes of neutrophils in response to a low-dose PMA and SSZ (Supplemental Fig. 5). We next attempted to determine the inhibitory effects of trolox on SSZ-induced NETosis *in vivo*. When injected s.c., SSZ provoked an inflammatory reaction and Ly-6G^+^ neutrophil accumulation *in situ* (Fig. 4i). Some of the accumulated neutrophils were immunoreactive for anti-citrullinated histone H3 antibodies (Fig. 4i), indicating they were undergoing NETosis. This acute inflammation resulted in the temporary swelling of the footpads of these mice (Fig. 4j). However, co-injecting trolox with SSZ substantially reduced the number of citrullinated histone H3-positive neutrophils, although neutrophils accumulated at a similar rate as was observed following the injection of SSZ injection (Fig. 4i). Consequently, injecting trolox significantly ameliorated footpad swelling in these mice (Fig. 4j). Finally, we evaluated the effects of trolox on NETosis in human neutrophils. Similar to the results observed in mouse neutrophils, trolox nearly completely inhibited low-dose PMA and SSZ- induced NETosis (Fig. 4k,l,m). These results indicate that SSZ enhances NETosis in activated neutrophils by accelerating lipid oxidation.

### Non-enzymatic oxidation of ether-linked phospholipids in neutrophils undergoing SSZ-induced NETosis

**Figure 5:**
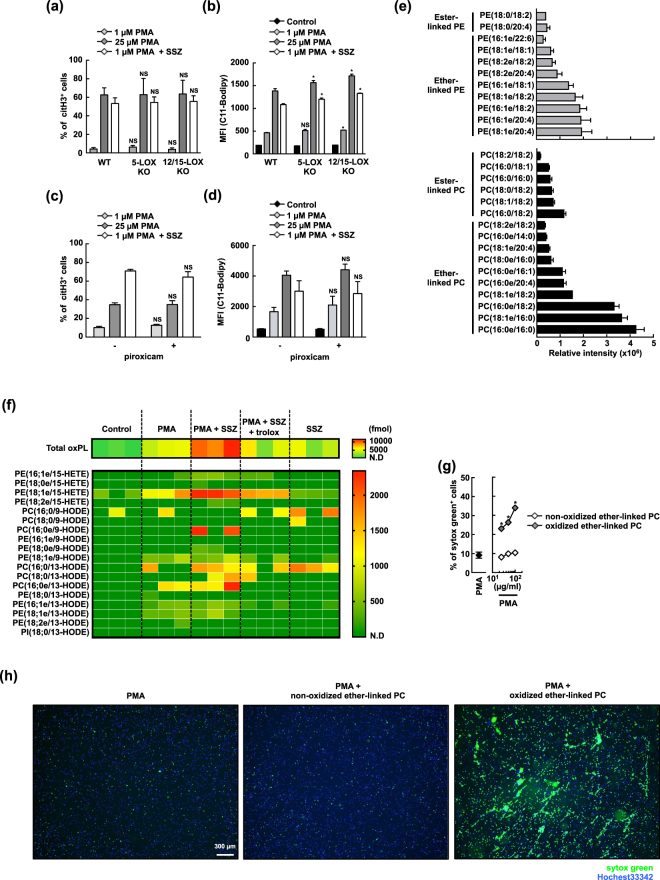
Non-enzymatic oxidation of ether-linked phospholipids in neutrophils undergoing NETosis in response to PMA and SSZ. (**a**,**b**) WT-, 5-LOX KO-, and 12/15-LOX KO-BM neutrophils were stimulated with 1 or 25 µM PMA alone, or 1 µM PMA + 1 mM SSZ for 4 h (**a**) or 2 h (**b**). (**a**) Cells were fixed and stain with DAPI and anti-citH3 polyclonal antibody. The proportion of cells undergoing NET formation was determined by counting the number of citH3^+^ cells using Image-J software. The values shown are averages and the s.d. of 3 or 4 mice per group. NS, not significant, two-way ANOVA, compared with WT. (**b**) C11-Bodipy^581/591^ was added to the cells, and the accumulation of lipid oxidation was analyzed using flow cytometry. Average mean fluorescent intensity (MFI) of C11-Bodipy analysis with s.d. of triplicated samples are shown. *P < 0.01, NS, not significant, two-way ANOVA, compared with WT. (**c**,**d**) BM neutrophils were stimulated with 1 or 25 µM PMA alone, or 1 µM PMA + 1 mM SSZ in the presence or absence of the non-selective COX inhibitor, 100 µM piroxicam for 4 h (**c**), or 2 h (**d**). (**c**) The proportion of cells undergoing NET formation was determined as described in (**a**). Average values and the s.d. of triplicated samples in a single experiment are shown. NS, not significant, two-way ANOVA, compared with piroxicam (−). The data shown are representative of two independent experiments. (**d**) The accumulation of lipid oxidation was analyzed as described in (**b**). Average mean fluorescent intensity (MFI) of C11-Bodipy analysis with s.d. of triplicated samples are shown. NS, not significant, two-way ANOVA, compared with piroxicam (−). The data shown are representative of two independent experiments. (**e**) Untargeted lipidomics of phospholipids in neutrophils. Lipid was extracted from naïve BM neutrophils isolated from WT mouse, and was analyzed by LC-MS/MS. Average and s.d. of three samples are shown. (**f**) Wide-targeted lipidomics of the oxidized phospholipids. Mouse BM neutrophils were stimulated with 1 µM PMA or/and 1 mM SSZ in the presence or absence of 400 µM trolox for 1 h. Lipid was extracted from these neutrophils, and wide-targeted analysis was performed using an ACQUITY UPLC system coupled with a triple quadrupole MS. (**g**,**h**) Oxidized ether-linked PC induces NETosis in activated mouse neutrophils. Mouse neutrophils were stimulated with 1 µM PMA. After 90 min, the cells were incubated with various concentrations of non-oxidized- or oxidized 1-O-hexadecyl-2-arachidonoyl-*sn*-glycero-3-phosphocholine (ether-linked PC) for 30 min. The cells were stained with sytox green and Hoechest33342. The frequencies of NETosis were determined by counting the number of sytox green^+^ cells (**g**), and visualized using fluorescence microscopy (**h**). (**g**) Average values and the s.d. of triplicated samples in a single experiment are shown. *P < 0.01, two-way ANOVA, compared with non-oxidized ether-linked PC. (**h**) Original magnification, × 20. The data shown are representative of two (**g**) or three (**h**) independent experiments.

Because lipid oxidation is responsible for accelerating SSZ-induced NETosis by SSZ, we hypothesized that SSZ may target a molecule that regulates lipid oxidation. In fact, it was previously reported that an oxidized fatty acid (5-hydroxyeicosatetraenoic acid (HETE)) that attaches to phospholipids as well as a free 5-HETE are generated by 5-lipoxygenase (5-LOX) in activated neutrophils^34^. We then sought to determine whether several enzymes involved in lipid oxidation are involved in PMA and SSZ-induced lipid oxidation. We first focused on LOX. Among several members of the mouse LOX gene family, we evaluated the involvement of 5-LOX and 12/15-LOX in SSZ-enhanced NETosis. Neutrophils were prepared from either 5-LOX-deficient^35^ or 12/15-LOX-deficient mice^36^ and were treated with a high dose of PMA alone or a low dose of PMA with SSZ. As shown in Fig. 5a, neutrophils obtained from either 5-LOX-deficient or 12/15-LOX-deficient mice underwent the same amount of NETosis that was observed in wild-type mice whether they were treated with a high dose of PMA alone or a low dose of PMA with SSZ. Consistent with these results, treatment with either a low dose of PMA and SSZ or a high dose of PMA efficiently enhanced lipid oxidation even when cells were deficient in 5-LOX or 12/15-LOX (Fig. 5b).

Cyclooxygenase (COX) is an enzyme known to be required for the oxidation of arachidonic acid. We next used piroxicam, a non-selective COX inhibitor, to evaluate whether COX is involved in SSZ-induced NETosis. We found that inhibiting COX did not have any effect on either the efficiency of NETosis induction (Fig. 5c) or PMA and SSZ-induced lipid oxidation (Fig. 5d).

These results strongly suggest that oxidized lipids are generated via non-enzymatic free radical attacks on lipids. Next, we attempted to determine what kind of oxidized lipids are actually generated during SSZ-induced NETosis. In these experiments, we performed a lipidomics analysis using lipid extraction on living and dying neutrophils followed by LC-MS/MS analysis. We first analyzed the composition of phospholipids in naïve neutrophils using untargeted lipidomics in an LC system coupled with a quadrupole/time-of-flight MS. We found that there was a significantly higher amount of ether-linked PC and PE than diacyl phospholipids (ester-linked PLs) in naïve neutrophils (Fig. 5e). We next sought to determine what molecular species of oxidized phospholipids were generated following stimulation with SSZ in PMA-activated neutrophils. In these experiments, we performed a wide-targeted analysis using an LC system coupled with a triple quadrupole MS. Consistent with the composition of phospholipids in naïve neutrophils (Fig. 5e), oxidized ether-linked phospholipids such as PE (18;1e/15-HETE) and PC (16;0e/13-HODE) were predominantly generated in neutrophils that were activated by PMA and SSZ (Fig. 5f), and the amount of these oxidized lipids was significantly decreased in cells treated with trolox (Fig. 5f). We further evaluated whether exogenous ether-linked oxidized lipids enhance NETosis in PMA-treated neutrophils. As shown in Fig. 5g and h, oxidized ether-linked PC significantly promoted NET formation in activated neutrophils. Taken together, these data led us to conclude that SSZ accelerates non-selective oxidation predominantly in ether-linked phospholipids and that these oxidized lipids are sufficient to induce NETosis. These results also imply that the oxidized lipids released from neutrophils undergoing NETosis may be capable of provoking the induction of NETosis in neighboring neutrophils.

### SSZ enhances NETosis via a different mechanism than ferroptosis

**Figure 6:**
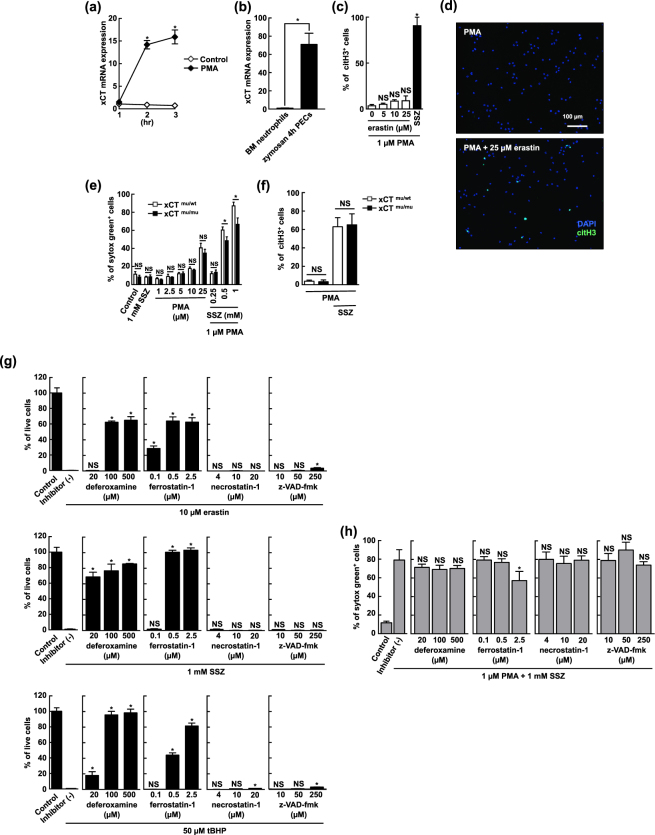
SSZ enhances NETosis via a different mechanisms than that used in ferroptosis. (**a**) xCT mRNA expression in PMA-stimulated mouse BM neutrophils. Cells were stimulated with 1 µM PMA for 1, 2, or 3 h. Total RNA was prepared from these cells and xCT mRNA expression levels were determined using qPCR. Expression levels were calculated as relative amounts and normalized to the levels of 18 s ribosomal RNA. The results are shown as the fold induction compared to the expression observed in naïve BM neutrophils. Average values and the s.d. of triplicated samples in a single experiment are shown. *P < 0.01, NS, not significant, two-way ANOVA. The data shown are representative of two independent experiments. (**b**) *In vivo* xCT mRNA expression in mouse peritoneal neutrophils. WT mice were intraperitoneally injected with 1 mg zymosan. After 4 h, the peritoneal cells were collected. Total RNA was prepared and xCT mRNA expression levels were determined using qPCR as described above. The average and s.d. of 3 mice are shown. *P < 0.05, Student’s t-test. (**c**,**d**) Erastin does not induce NET formation. Mouse neutrophils were stimulated with 1 µM PMA in the presence or absence of various concentrations of erastin or 1 mM SSZ. The proportion of cells undergoing NET formation were measured by counting the number of citH3^+^ (**c**) and visualized by staining of DAPI and citH3 (**d**). Average values and the s.d. of triplicated samples in a single experiment are shown (**c**). Original magnification, ×20 (**d**). *P < 0.01, NS, not significant, one-way ANOVA, compared with 1 µM PMA. The data shown are representative of three independent experiments. (**e**,**f**) NET formation was observed in xCT-mutant neutrophils. BM neutrophils obtained from xCT^mu/wt^ or xCT^mu/mu^ mice were stimulated with PMA in the presence or absence of SSZ. The frequencies of cells undergoing NET formation were measured by counting the number of sytox green^+^ (**e**) or citH3^+^ cells (**f**). Average values and the s.d. of triplicated samples in a single experiment are shown. *P < 0.01, NS, not significant, two-way ANOVA. The data shown are representative of two (**e**) or three (**f**) independent experiments. (**g**) NIH3T3 cells were treated with erastin, SSZ, or tBHP in the presence or absence of ferroptosis inhibitors (deferoxamine and ferrostatin-1), a necroptosis inhibitor (necrostatin-1), or an apoptosis inhibitor (z-VAD-fmk). Cell viability was assessed using WST-8 assays according to the manufacturer’s protocols. Average values and the s.d. of triplicated samples in a single experiment are shown. *P < 0.01, NS, not significant, one-way ANOVA compared with inhibitor (−). The data shown are representative of two independent experiments. (**h**) Mouse neutrophils were treated with PMA and SSZ in the presence or absence of ferroptosis inhibitors, a necroptosis inhibitor, or apoptosis inhibitor. NET formation was evaluated as described in (**e**). Average values and the s.d. of triplicated samples in a single experiment are shown. NS, not significant, one-way ANOVA compared with inhibitor (−). The data shown are representative of two independent experiments.

SSZ is reported to induce ferroptosis in cancer cells by inhibiting the function of xCT^37,38^. xCT is expressed in a variety of cell types and plays a crucial role in the maintenance of intracellular glutathione levels by transporting cystine into the cells. Thus, inhibiting xCT causes glutathione to be depleted in cells, which increases cellular fragility in response to oxidative stress. In fact, the expression level of the xCT transcript was increased in neutrophils that were stimulated with PMA *in vitro* (Fig. 6a) or with zymosan *in vivo* (Fig. 6b). Following on these results, we sought to determine whether xCT is involved in accelerating SSZ-induced NETosis. In these experiments, we first investigated the effects of erastin, which is another inducer of ferroptosis that acts by inhibiting xCT, on NETosis. As shown in Supplemental Fig. 6, less than 1 µM of erastin was capable of inducing cell death in NIH3T3 cells, and 200 µM of SSZ was required to kill all of the cells in 12 h, indicating that erastin is a very potent inducer of ferroptosis in NIH3T3 cells. However, erastin failed to accelerate NETosis in mouse neutrophils that were treated with a low dose of PMA (Fig. 6c,d). We next analyzed the effects of SSZ on xCT-deficient neutrophils obtained from xCT-mutant mice^39^. In these mice, N-ethyl-N-nitrosourea (ENU) mutagenesis caused the premature termination of the xCT gene, resulting in a loss-of-function mutation in xCT. Embryonic fibroblasts and bone marrow-derived macrophages obtained from these mice did not survive or proliferate *in vitro* without 2-ME. We first evaluated NETosis by PMA alone with sytox green in neutrophils that were prepared from these mice. We found that there was no difference in the efficiency with which NETosis was induced by PMA alone between WT and xCT mutant neutrophils (Fig. 6e). Moreover, SSZ accelerated NETosis in activated xCT mutant neutrophils, although they were slightly resistant to this stimulus compared with those from WT mice (Fig. 6e). We also evaluated NETosis by PMA + SSZ with anti-citH3 Ab, and found that SSZ accelerated NETosis in activated xCT mutant neutrophils and those obtained from WT mice to the same degree (Fig. 6f). These results clearly indicate that xCT is not a target molecule of SSZ because it does not affect the acceleration of NETosis by SSZ in activated neutrophils.

The generation of oxidized lipid is also involved in ferroptosis, suggesting that some cell death signaling mechanisms may be shared between SSZ-induced NETosis and ferroptosis. Thus, we next sought to identify the differences between the mechanisms involved in ferroptosis and SSZ-induced NETosis by comparing the effects of several cell death inhibitors on each type of cell death. In NIH3T3 cells, erastin or SSZ-inducing ferroptosis was effectively inhibited by deferoxamine or ferrostatin-1, while neither necrostatin-1 nor z-VAD was effective in blocking ferroptosis (Fig. 6g). Deferoxamine and ferrostatin-1 also inhibited tert-butyl hydroperoxide (tBHP)-induced ferroptosis-like cell death in NIH3T3 cells. On the other hand, deferoxamine never inhibited PMA + SSZ-induced NETosis (Fig. 6h). Only a high dose (2.5 μM) of ferrostatin-1 partially inhibited NETosis by PMA + SSZ, but it was less effective compared with the case of ferroptosis in NIH3T3 cells (Fig. 6h).

Taken together, these data indicate that while lipid oxidation is involved in both SSZ-induced NETosis and ferroptosis signaling, these two cell death pathways do not share common mechanisms downstream of lipid oxidation.

### The structure-activity relationship between NET-inducing compounds

**Figure 7:**
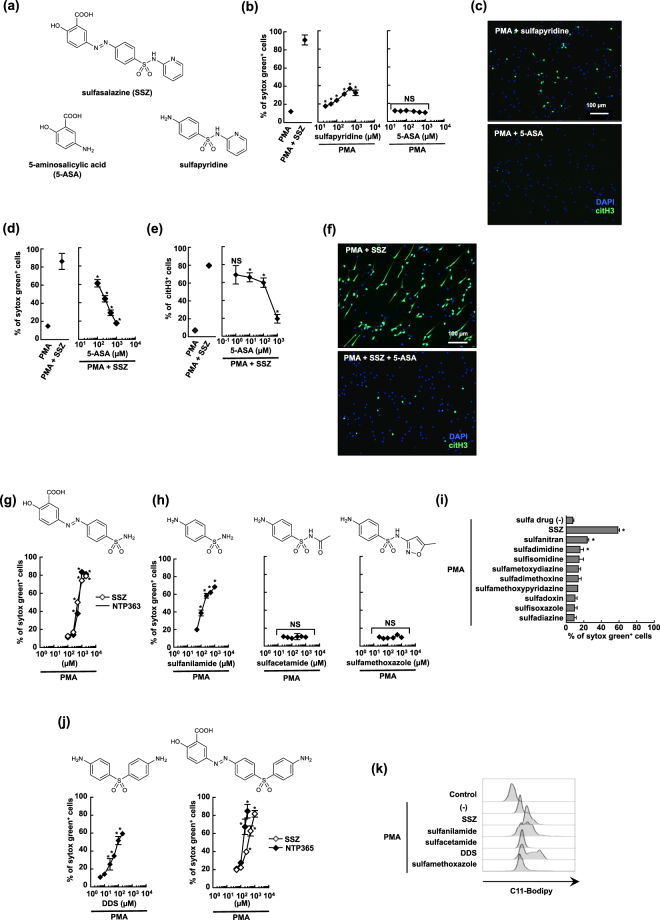
The structure-activity relationship between NET-inducing compounds. (**a**) The structures of SSZ, 5-aminosalicylic acid (5-ASA), and sulfapyridine. (**b**,**c**) Sulfapyridine, but not 5-ASA, induces NETosis. Mouse BM neutrophils were stimulated with various concentration of sulfapyridine or 5-ASA in the presence of 1 µM PMA for 4 h. The proportion of cells undergoing NET formation was determined by counting the number of sytox green^+^ cells (**b**) and NET formulation was visualized by staining of DAPI and anti-citH3 Ab (**c**). (**b**) Average values and the s.d. of triplicated samples in a single experiment are shown. *P < 0.01, NS, not significant, one-way ANOVA, compared with PMA. (**c**) Original magnification, ×20. The data shown are representative of two independent experiments. (**d**–**f**) 5-ASA inhibits SSZ-induced NETosis. Mouse neutrophils were stimulated with 1 µM PMA alone, 1 µM PMA + 1 mM SSZ with or without various concentrations of 5-ASA. NET formation was evaluated with sytox green (**d**), or anti-citH3 Ab (**e**,**f**). (**d**,**e**) Average values and s.d. of triplicated samples in a single experiment are shown. *P < 0.01, NS, not significant, one-way ANOVA, compared with PMA + SSZ. (**f**) Original magnification, ×20. The data shown are representative of two independent experiments. (**g**–**j**) Mouse neutrophils were stimulated with 1 µM PMA in the presence of various concentrations of SSZ, NTP363 (**g**), sulfa drugs (sulfanilamide, sulfacetamide, and sulfamethoxazole) (**h**), 1 mM various sulfa drugs having a variety of substituent on the sulfonamides (**i**), DDS (**j**, left), SSZ, or NTP365 (**j**, right) for 4 h. The proportion of cells undergoing NET formation was measured using sytox green. Average values and the s.d. of triplicated samples in a single experiment are shown. *P < 0.01, NS, not significant, one-way ANOVA, compared with PMA alone. The data shown are representative of two independent experiments. (**k**) Mouse neutrophils were stimulated with 1 µM PMA in the presence of 1 mM SSZ, or sulfa drugs (1 mM sulfanilamide, 1 mM sulfacetamide, 100 µM DDS, and 1 mM sulfamethoxazole) for 1 h. C11-Bodipy^581/591^ was then added, and the accumulation of lipid oxidation was analyzed. The data shown are representative of two independent experiments.

It is widely reported that SSZ is converted to 5-aminosalicylic acid (5-ASA) and sulfapyridine by bacterial azo-reductase in the colon^40^ (Fig. 7a). It is thought that 5-ASA contributes to anti-inflammatory activity of SSZ and that sulfapyridine might be responsible for some of the side effects^41^. Thus, we next examined the effects of these SSZ metabolites on NETosis. As shown in Fig. 7b and c, sulfapyridine enhanced PMA-induced NETosis, though the effect was much weaker than that of SSZ. In contrast, 5-ASA did not enhance NETosis. Furthermore, interestingly, 5-ASA inhibited PMA and SSZ-induced NETosis in a dose-dependent manner (Fig. 7d,e and f). These results may explain why the incidence of SSZ-induced agranulocytosis remains very low when it is orally administered.

To further clarify the structure-activity relationship of SSZ, we first synthesized an SSZ drivative lacking the pyridyl group (NTP363, previously reported as salazosulfamide), and tested its effect on NET formation. NTP363 showed comparable activity to SSZ, indicating that the pyridyl group is not essential for its NETosis-enhancing activity (Fig. 7g). Since sulfapyridine belongs to the antimicrobial sulfa drugs, we next evaluated the NETosis-enhancing activity of other known sulfa drugs. As a result, sulfanilamide, which is reported to cause DIAG^42^, was found to show stronger NETosis-enhansing activity than sulfapyridine, while both sulfacetamide and sulfamethoxazole showed no activity (Fig. 7h). We also checked other sulfa drugs having a variety of substituents on the sulfonamides (Structure are shown in Supplemental Fig. 7), and found that no sulfa drugs other than sulfanilamide possessed such activity (Fig. 7i). To know whether the sulfonamide structure is essential for the NETosis-enhancing activity, we also tested 4,4′-diaminodiphenyl sulfone (DDS), which is reported to show similar antibacterial activity to sulfa drugs, and also to cause DIAG^43^. DDS showed similar NETosis-enhancing activity to sulfanilamide at one order of magnitude lower dose (Fig. 7j, left). We also synthesized a sulfone derivative of SSZ, NTP365, in which the sulfonamide group was replaced with 4-aminophenyl sulfone. NTP365 exhibited stronger activity than SSZ (Fig. 7j, right). These results suggest the future possibility of developing advanced NETosis-enhancer molecules based on DDS structure.

In line with their levels of NETosis-enhancing activity, the NETosis-inducing compounds also accelerated lipid oxidation in PMA-treated neutrophils (Fig. 7k). These results support the notion that sulfanilamide and sulfone derivatives induce NETosis in the same manner as SSZ.

## Discussion

In this study, we showed that SSZ accelerates the oxidation of phospholipids in activated neutrophils, which consequently enhances NETosis. These findings, together with recent studies of ferroptosis, shed light on the role of oxidized phospholipids as executors or modulators of specific types of cell death. In the case of ferroptosis, it has been reported that glutamate or other small compounds that inhibit the Xc^−^ cystine transporter system cause GSH depletion, which results in the loss of cellular anti-oxidant capacity and cellular fragility in response to ROS^44^. Small compounds that inhibit GPX4, a unique cellular enzyme involved in reducing oxidized lipids, also induced ferroptosis, indicating that lipid oxidation plays critical roles in this type of cell death. We initially thought that SSZ augmented lipid hyperoxidation by inhibiting the function of xCT because this was the mechanism that is believed to underlie ferroptosis. However, we concluded that SSZ promotes the accumulation of oxidized lipids and consequently induces NETosis in an xCT-independent manner. In addition, our data led to the two following findings. First, ferroptosis inhibitors did not affect SSZ-induced NETosis. Second, SSZ-induced NETosis did not require the enzymatic activity of 12/15 LOX, which is indispensable to the induction of ferroptosis in GPX4-deficient cells^45,46^. All of these findings support the idea that SSZ-induced NETosis is distinct from the cell death process involved in ferroptosis, although both types of cell death involve lipid oxidation. At this moment, the molecule that SSZ targets to induce lipid oxidation and the subsequent induction of NETosis remains unknown. One possibility is that SSZ inhibits the ability of this molecule to reduce oxidized phospholipids. Alternatively, SSZ may positively regulate unidentified molecules that are involved in lipid oxidation. In any case, we expect that identifying the molecule targeted by SSZ should facilitate our ability to characterize the molecular paradigms that underlie the regulation of NETosis and NET formation.

Because the integrity of the plasma membrane is disrupted during NETosis, it is conceivable that intracellular materials are released from neutrophils undergoing NETosis. In this study, we found that exogenous oxidized phospholipids were capable of rapidly inducing NETosis. This finding strongly suggests that the release of these oxidized phospholipids induces the sequential activation of NETosis in neighboring neutrophils. This NETosis chain reaction may contribute to the effective formation of NETs in infected sites. In addition to the roles played by intracellular signaling molecules, lipids have also been reported to perform myriad and diverse extracellular functions. Among the wide variety of lipid molecular species, the roles of oxidized phospholipids have received an especially large amount of attention. For instance, it has been reported that exogenous oxidized phospholipids control oxidative bursts in neutrophils^47^ and inhibit Toll-like receptor signaling^48^, indicating that they have anti-inflammatory capabilities. On the other hand, the results of a recent study demonstrated that oxidized phospholipids can bind and activate caspase-11 and elicit the release of IL-1 from dendritic cells^49^. In consideration of these previous studies, we suggest that it is possible that the oxidized phospholipids released during NETosis play multiple roles in immune regulation during tissue damage and inflammation.

In this study, we demonstrated that ether-linked oxidized phospholipids induced NETosis in neutrophils. However, they did not induce any toxic effects in NIH3T3 cells, at least when the cells were treated with these oxidized phospholipids for short periods of time (4 h). These findings strongly suggest that a specific mechanism underlies the ability of neutrophils to response to oxidized lipids. Neutrophils may possess a unique ability to incorporate oxidized lipids into their cytosol. Alternatively, neutrophils may express a cell surface receptor that responds to oxidized lipids. For instance, platelet-activating factor (PAF) is an ether-linked lysophospholipid that exerts diverse functions via the PAF receptor. It was previously reported that certain oxidized phospholipids that are structurally related to PAF can be generated in a non-enzymatic manner and that they act as agonists at the PAF receptor^50–53^. In any case, elucidating the mechanism by which oxidized lipids induce NETosis will substantially increase our ability to understand the overall purpose of and processes underlying this type of cell death.

Idiosyncratic drug-induced agranulocytosis (DIAG) is one of the most serious and potentially life-threatening adverse effects of a wide variety of drugs^54^. Among these drugs, SSZ has frequently been reported to be involved in DIAG. Many hypotheses have been proposed to explain the pathogenesis of DIAG, but it remains elusive. One of the possible mechanisms by which DIAG may act involves the modification of causative drugs to generate toxic metabolites^55^. Some drugs have been reported to be oxidized by the ROS generated by activated neutrophils, and these oxidized compounds may exert toxic effects on neutrophils^56,57^. In this context, it is likely that neutrophils exposed to these toxic effects undergo a specific mode of cell death. Additionally, in the case of SSZ, oxidative stress has been speculated to be involved in its cytotoxicity^58^. However, the details underlying the mechanisms involved in toxicity in neutrophils remain unknown. In this manuscript, we have demonstrated that in activated neutrophils, the SSZ-induced accumulation of oxidized phospholipids caused the acceleration of NETosis. This excessive NETosis is inhibited by trolox, a water soluble analogue of vitamin E. Although the mechanisms by which SSZ promotes lipid oxidation remain enigmatic at this point, the findings described in this study indicate that there are potentially beneficial effects of inhibiting lipid oxidation as a preventive or therapeutic treatment for SSZ-induced DIAG. Evidence is accumulating that indicates that some drugs affect NETosis or NET formation, and consequently provoke autoimmune responses or vasculitis. For example, propylthiouracil, an antithyroid drug, induced conformational changes in PMA-induced NET, the production of anti-neutrophil cytoplasmic antibodies, and subsequent vasculitis^59^. Very recently, levamisole, a drug currently used to treat parasitic infections in veterinary medicine, was found to induce NET formation in neutrophils, and this mechanism is potentially implicated in drug-induced autoimmunity and vasculitis^60^. It is noteworthy that this drug also causes DIAG, suggesting that there is a relationship between excessive NETosis or NET formation and agranulocytosis. Moreover, sulfanilamide and DDS, which exerted NETosis-promoting activity in our study, are also known to cause DIAG^42,43^. Our and others’ findings strongly suggest that accelerating NET formation is one of the causes of DIAG.

In conclusion, in this manuscript, we have shown that SSZ enhances NETosis by accelerating lipid oxidation. Our analysis of the relationship between the structure and activity of SSZ and its related compounds will enable us to develop chemical compounds that possess potent NET-inducing activity by accelerating lipid oxidation. These results clearly demonstrate that hyperoxidation of phospholipids is a key mechanism to accelerate NET formation. These findings expand our understanding of the development of NET-regulating compounds.

## Methods

### Animals and human donor-derived peripheral blood

C57BL/6 J mice were purchased from CLEA Japan. xCT^mu/mu^ mice (C57BL/6 background) were previously generated by our laboratory^39^. The 5-LOX^35^ and 12/15-LOX KO mice^36^ (C57BL/6 background) were purchased from Jackson laboratory. All experiments using mice were approved by the Tokyo University of Pharmacy and Life Sciences Animal Care Committee (L16-14) and performed in accordance with applicable guidelines and regulations. The use of healthy human donor-derived peripheral blood was approved by the human ethics committee of Tokyo University of Pharmacy and Life Sciences (15–21) and all methods were performed in accordance with ethical guidelines for medical and health research involving human subjects in Japan. All blood donors gave informed consent.

### Reagents

Deferoxamine, ferrostatin-1, erastin, sulfacetamide, sulfasalazine (SSZ), zymosan, diphenyleneiodonium chloride (DPI), 4,4′-diaminodiphenyl sulfone (DDS), sulfisoxazole, tBHP, and sulfasalazine were purchased from Sigma. Phorbol 12-myristate 13-acetate (PMA), sulfamethoxazole, sulfanilamide, sulfapyridine, 5-aminosalicylic acid (5-ASA), sulfadimidine, sulfametoxydiazine, sulfisomidine, sulfanitran, sulfanilamide, and piroxicam were purchased from Wako. We purchased 1-O-hexadecyl-2-arachidonoyl-*sn*-glycero-3-phosphocholine (ether-linked phosphatidylcholine) from Avanti polar lipids. Necrostatin-1 was purchased from FOCUS Biomolecules, and 2-mercaptoethanol was purchased from MP Biomedicals. z-VAD-fmk was purchased from Peptide institute. Trolox, sulfadiazine, sulfadimethoxine, sulfadoxin, and sulfamethoxypyridazine were purchased from Tokyo Chemical Industry. Cl-amidine was purchased from Cayman chemicals. Ionomycin was purchased from Merck Millipore.

### Generation of monoclonal antibodies

To generate anti-histone H3 (citrulline R2 + R8 + R17) antibodies, a Wistar rat was subcutaneously immunized in the foot pad with a peptide derived from human histone H3 (citrulline R2 + R8 + R17) (2–20: A(cit)RTKQTA(cit)RKSTGGKAP(cit)RKQ) emulsified in adjuvant (TiterMax Gold; TiterMax). Splenocytes were fused with NSO^bcl2^ myeloma cells^61^ using PEG1500 (Roche, Germany). Hybridoma cells were selected in DMEM/10% FCS containing HAT (Sigma) and 5% BM-Condimed (Roche). The hybridoma supernatants were tested using ELISA, and a monoclonal antibody designed to specifically detect human histone H3 (citrulline R2 + R8 + R17) peptides but not human non-citrullinated histone H3 peptides was obtained (11-11B-4F). The IgG fraction was purified using a Protein G affinity column (GE Healthcare), and the buffer exchange to PBS was performed using PD-10 columns (GE Healthcare). The antibody was biotinylated using EZ-Link Sulfo-NHS-LC-LC-Biotin (Thermo Fisher Scientific) according to the manufacturer’s recommendations.

### Mouse and human neutrophil isolation

To obtain mouse neutrophils, BM cells were isolated from the femurs and tibias of C57BL/6 J WT, KO, and mutant mice. The BM cells were incubated with Fc blocker (93; Biolegend) and stained with biotinylated anti-Ly-6G (RB6-8C5; Biolegend) antibodies. They were then incubated with anti-biotin-microbeads (Miltenyi Biotech). Ly-6G^high^ cells were enriched using magnetic sorting. The purity of the isolated neutrophils was more than 95% when assessed using flow cytometry (FACSverse; BD).

To obtain human neutrophils, peripheral blood was collected from healthy adult volunteers using heparin. Red blood cells were removed using HetaSep^TM^ (STEMCELL Technologies) sedimentation according to the manufacturer’s protocol. Then, the cells were washed twice with RPMI 1640 medium and further fractionated on a discontinuous Percoll PLUS (GE-healthcare) gradient that consisted of layers with densities of 75%, 65%, and 55%. After the mixture was centrifuged for  30 min at 500 × g, the interface between the 65% and 75% layers was collected and washed twice with RPMI 1640 medium. All procedures were conducted at room temperature. The preparations contained more than 95% of CD15^+^CD16^+^Siglec-8^−^ neutrophils according to flow cytometric analysis. Cell viability was >98% according to trypan blue exclusion assays.

### Cells and cell culture

NIH3T3 fibroblasts were maintained in Dulbecco’s modified Eagle’s medium (DMEM, WAKO) supplemented with 10% fetal bovine serum (GIBCO) and 1% penicillin-streptomycin at 37 °C, 5% CO2, and 95% humidity.

### *In vitro* cell death assay

To detect SSZ-induced apoptosis and necrosis in isolated neutrophils, 1.4 × 10^4^ mouse neutrophils were incubated with SSZ. After 4 or 12 h, the cells were stained with FITC-Annexin V and 7-AAD (Biolegend). A flow cytometric analysis was then performed using a BD FACSverse.

To assess NETosis in isolated mouse or human neutrophils, 4 × 10^5^ neutrophils were seeded in a 35-mm ploy-_L_-lysine-coated glass bottom dish (MATSUNAMI) and stimulated with PMA and/or SSZ for 2 or 3.5 h. Then, sytox green (0.5 µM, Thermo Fisher Scientific) and/or Hoechest 33342 (1 µg/ml, Thermo Fisher Scientific) were added to the cells. After 30 min, the frequency of NETosis was measured by counting the number of sytox green^+^ cells using an IN Cell Analyzer 2000 (GE Healthcare), Operetta CLS (PerkinElmer) or Image-J software (NIH), and a morphological analysis was performed using a fluorescence microscope (BZ-X710, Keyence).

To detect the citrullination of histone H3 in mouse neutrophils, 4 × 10^5^ cells were seeded in a 35-mm ploy-_L_-lysine-coated glass bottom dish (MATSUNAMI) and stimulated with PMA, ionomycin, and/or SSZ for 4 h. The cells were then fixed with 4% paraformaldehyde for 10 min at room temperature and incubated in HBSS supplemented with 10% normal goat serum (Sigma), bovine serum albumin (Sigma) and 0.01% Tween 20 for 1 h for blocking. The cells were then incubated first with rabbit anti-histone H3 (citrulline R2 + R8 + R17) (citH3) antibodies (Abcam) and then with anti-rabbit IgG antibodies coupled with Alexa Fluor 488 (Thermo Fisher Scientific). DNA was labeled using DAPI (DOJINDO). We observed the cells under a fluorescence microscope (BZ-X710) and quantified the frequency of cells undergoing NETosis by measuring the number of citH3^+^ cells using Image J software.

To detect the amount of histone H3 citrullination in human neutrophils, 4 × 10^5^ cells were seeded in a 35-mm ploy-_L_-lysine-coated glass bottom dish (MATSUNAMI) and stimulated with PMA for 2.5 h. The cells were fixed and blocked and then incubated first with biotinylated anti-citH3 Ab (11-11B-4F) and then streptavidin coupled with Cy3 (Jackson lab). We quantified the frequency of cells undergoing NETosis as described above.

To assess the viability of NIH3T3 cells, the cells were incubated with tBHP, erastin, or SSZ in the presence of ferroptosis inhibitors (e.g., deferoxamine or ferrostatin-1), a necroptosis inhibitor (necrostatin-1), or an apoptosis inhibitor (z-VAD-fmk). After 12 h, cell viability was determined using a Cell Counting kit-8 (DOJINDO) according to the manufacture’s methods.

### Detection of neutrophil cell death *in vivo*

To determine the number of neutrophils in the peripheral blood or in the peritoneal cavity, mice were intraperitoneally injected with 16 mg SSZ and/or 1 mg zymosan. After 4 or 24 h, peripheral blood cells or peritoneal cells were collected, and a red blood cell lysis was performed using Pharm Lyse (BD Biosciences) to obtain leukocytes. The leukocytes were stained with PE-conjugated anti-Ly-6C, PE-Cy7-conjugated anti-CD45.2, APC-Cy7-conjugated anti-Ly-6G antibodies, and 7-AAD. The absolute number of CD45.2^+^Ly-6C^-^Ly-6G^+^ neutrophils and the percentage of dead cells (7-AAD^+^ cells) were determined by counting the relevant cells on a microscope using a hemocytometer and analyzing the cell numbers using flow cytometry on a BD FACSverse.

To detect the citrullination of histone H3, peritoneal cells were placed on glass slides using Cytospin 4 (Thermo Fisher Scientific) and stained as described above.

To detect the citrullination of histone H3 in neutrophils in mouse footpads, mice were subcutaneously injected with SSZ with or without trolox. After 48 h, the footpads were resected and embedded in OCT compound (Sakura) and frozen in cooled hexane. Sections were generated using Kawamoto’s film method^62^ (Leica). We prepared 5-µm-thick cryosections, which were air-dried and fixed in 100% ethanol and 4% paraformaldehyde. The sections were then stained first with Alexa647-conjugated anti-Ly-6G and rabbit anti-citH3 antibodies (Abcam) with anti-rabbit IgG antibodies coupled with Alexa Fluor 488 (Thermo fisher). DNA was labeled using DAPI. We observed the cells under a fluorescence microscope.

### Detection of intracellular ROS generation and lipid oxidation

To detect the intracellular generation of ROS, 1.4 × 10^4^ mouse neutrophils were incubated in the presence of 10 µM DCFH-DA (Invitrogen) in Hank’s balanced salt solution (HBSS (+)) at 37 °C for 5 min and then stimulated using PMA or ionomycin in the presence or absence of SSZ at 37 °C for 15 min. A flow cytometric analysis was perfumed using a BD FACSverse.

To detect lipid oxidation, 1.4 × 10^4^ mouse neutrophils were seeded in round-bottom 96-well plates and stimulated with PMA or ionomycin in the presence of SSZ or sulfa drugs. After 60 min, 2 µM C11-Bodipy^581/591^ (Thermo Fisher Scientific) was added to the cells for 30 min. A flow cytometric analysis was perfumed using a BD FACSverse.

### Lipid extraction from neutrophils

5 × 10^6^ mouse neutrophils were harvested in ice-cold methanol, and lipids were extracted using solid-phase extraction in a monospin C18 column (GL Sciences). PC (17:0/14:1), PE (17:0/14:1), PI (17:0/14:1), PS (17:0/14:1) and PG (17:0/14:1) were added at final concentration of 100 nM each and used as the internal standard. The extracted lipids were reconstituted in 40 μl of chloroform:methanol = 1:2 and stored at −80 °C until used.

### Untargeted lipidomics

An untargeted lipidomics analysis was performed using an ACQUITY UPLC system (Waters, Milford, MA) coupled with a quadrupole/time-of-flight MS (TripleTOF 6600, Sciex, Framingham, MA). The LC separation was performed using a reverse-phase column (AQUITTY UPLC BEH peptide C18 (50 mm × 1.7 mm inner diameter, 2.1 μm particle size; Waters) with a gradient elution in mobile phase A (methanol/acetonitrile/water = 1:1:3 v/v/v with 5 mM ammonium acetate (Wako Chemicals, Osaka, Japan) and 10 nM EDTA (DOJINDO) and mobile phase B (100% isopropanol containing 5 mM ammonium acetate and 10 nM EDTA). The LC gradient consisted of holding solvent (A/B:100/0), which was applied for 1 min. It was linearly converted to solvent (A/B:60/40) over 4 min, linearly converted to solvent (A/B:36/64) over 2.5 min, held for 4.5 min, linearly converted to solvent (A/B:17.5/82.5) for 0.5 min, and linearly converted to solvent (A/B:5/95) for 1 min before being returned to solvent (A/B:100/0) and held for 5 min for re-equilibration. The injection volume was 2 μl, the flow rate was 0.300 ml/min, and the column temperature was 45 °C. Information dependent acquisition (IDA) mode was applied to confirm the biogenic phospholipid structures. The ionization efficiency was normalized by using standards of ester-linked and ether-linked PC. The peak alignment and picking were performed using 2-DICAL^63^ (Mitsui Knowledge Industry).

### Wide-targeted analysis

A wide-targeted analysis was performed using an ACQUITY UPLC system (Waters, Milford, MA) coupled with a triple quadrupole MS (QTRAP 6500, Sciex). The LC separation was performed using a reverse-phase column [ACQUITY UPLC HSS T3 (50 mm × 2.1 mm inner diameter, 1.8 μm particle size; Waters)] with a gradient elution consisting of mobile phase A (methanol/acetonitrile/water = 1:1:3 v/v/v containing 50 mM ammonium acetate and 10 nM EDTA) and mobile phase B (100% isopropanol containing 50 mM ammonium acetate and 10 nM EDTA). The LC gradient consisted of holding solvent (A/B:100/0) for 1 min, which was linearly converted to solvent A/B:50/50 for 4 min, linearly converted to solvent A/B:36/64 for 7 min, linearly converted to solvent A/B:5/95 for 1 min and then held for 1 min. It was then returned to solvent A/B:100/0 and held for 5 min for re-equilibration. The injection volume was 3.5 μl, the flow rate was 0.350 ml/min, and the column temperature was 50 °C. Multiple reaction monitoring (MRM) mode was used to quantify oxidized phospholipids in biological samples.

### Quantitative RT–PCR

Total RNA was extracted from neutrophils using an RNeasy Mini kit (QIAGEN) according to the manufacturer’s instructions. Complementary DNAs were synthesized using a ReverTra Ace qPCR Master Kit (TOYOBO). qPCR was performed on complementary DNA using THUNDERBIRD SYBR qPCR Mix (TOYOBO). The reactions were run using a real-time PCR system (StepOne Plus, Applied Biosystems). The expression levels were normalized to 18 s ribosomal RNA and are displayed as the fold induction over naïve controls unless otherwise stated. The following PCR primers were used for xCT (SLC7a11): xCT-F, 5′AGAGCATCACCATCGTCAGA3′ and xCT-R, 5′GATTCATGTCCACAAGCACAC3′; for 18 s ribosomal RNA were: 18 s ribosomal RNA-F, 5′CGGACAGGATTGACAGATTG3′ and 18 s ribosomal RNA-R, 5′CAAATCGCTCCACCAACTAA3′.

### Electron microscopy

Mouse neutrophils obtained from WT mice were cultured on ploy-_L_-lysine-coated cover glass (MATSUNAMI) and stimulated with PMA and/or SSZ. After 1 or 2 h, the cells were fixed by a conventional method (1.5% paraformaldehyde and 3% glutaraldehyde in 0.1 M phosphate buffer, pH 7.4, followed by an aqueous solution of 1% osmium tetroxide). Fixed samples were embedded in Epon 812, and thin sections (70–80 nm) were then cut and stained with uranyl acetate and lead citrate for observation under a Jeol-1010 electron microscope (Jeol) at 80 kV.

### Preparation of oxidized ether-linked phosphatidylcholine

Ether-linked phosphatidylcholine (PC) in chloroform was evaporated to dryness in a glass tube and desiccated for at least 1 h *in vacuo*. Dry ether-linked PC was oxidized in an air incubator at 37 °C for 48 h. Oxidized ether-linked PC was hydrated by adding serum-free medium (Advanced RPMI1640; Thermo Fisher Scientific), vortexed at room temperature for 5 min, and then sonicated for 2 min.

### Synthesis of NTP compounds

Detailed experimental procedures for the preparation of new NTP compounds are provided in Supplemental information.

### Statistical analysis

Paired- and unpaired two-tailed Student’s t-tests and Mann-Whitney U-tests were used to compare two groups. One- and two-way ANOVA was used to compare multiple groups. All statistical analyses were performed using Graph Pad Prism 7 software.

## Electronic supplementary material

supplementary information
