# Supplementary material for: Hyperoxidation of ether-linked phospholipids accelerates neutrophil extracellular trap formation
Source: Sci Rep. 2017 Nov 22;7:16026. doi: 10.1038/s41598-017-15668-z (PMC5700140; doi:10.1038/s41598-017-15668-z)
Supplement: Supplementary file 1 — supplementary information [file 41598_2017_15668_MOESM1_ESM.pdf]

<u>SUPPLEMENTARY INFORMATION</u>

**Hyperoxidation of ether-linked phospholipids accelerates neutrophil extracellular trap formation**

Satoshi Yotsumoto[1], Yuito Muroi[1], Tatsuya Chiba[1], Rio Ohmura[1], Maki Yoneyama[1], Megumi Magarisawa[1], Kosuke Dodo[2], Naoki Terayama[2], Mikiko Sodeoka[2], Ryohei Aoyagi[3, 4], Makoto Arita[3, 4, 5], Satoko Arakawa[6], Shigeomi Shimizu[6] and Masato Tanaka[1, *]

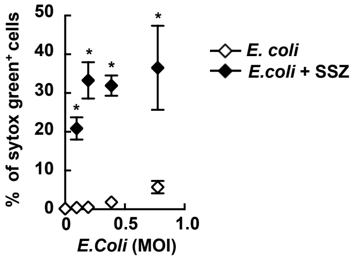


**Supplemental Figure. 1.** Human peripheral blood neutrophils were stimulated with *E. coli* (strain XL1-Blue) in the presence or absence of 1 mM SSZ for 2.5h. Cells were stained with sytox green. The proportion of dead cells was determined by counting the number of sytox green$^{+}$ cells using Operetta CLS. Average values and the s.d. of triplicated samples in a single experiment are shown. *$P < 0.001$, two-way ANOVA, compared with *E.coli*. The data shown are representative of two independent experiments.

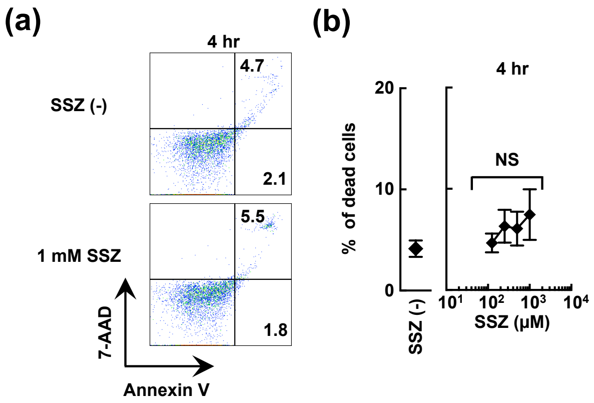


**Supplemental Figure. 2.** (a, b) Mouse neutrophils were incubated with or without various concentration of SSZ for 4h. (a) Flow cytometric analysis of cell death in SSZ-treated neutrophils. Dead cells were detected using double labeling with 7-AAD and Annexin V by flow cytometry. The numbers in the dot plots indicate the frequencies of 7-AAD and/or Annexin V-positive cells. (b) The proportion of dead cells was quantified using the sum of percentage of 7-AAD and/or Annexin V-positive cells at 4h after SSZ treatment. Average values and the s.d. of triplicated wells of single experiment are shown. NS, not significant, one-way ANOVA, compared with SSZ (-). Data are representative of two independent experiments.

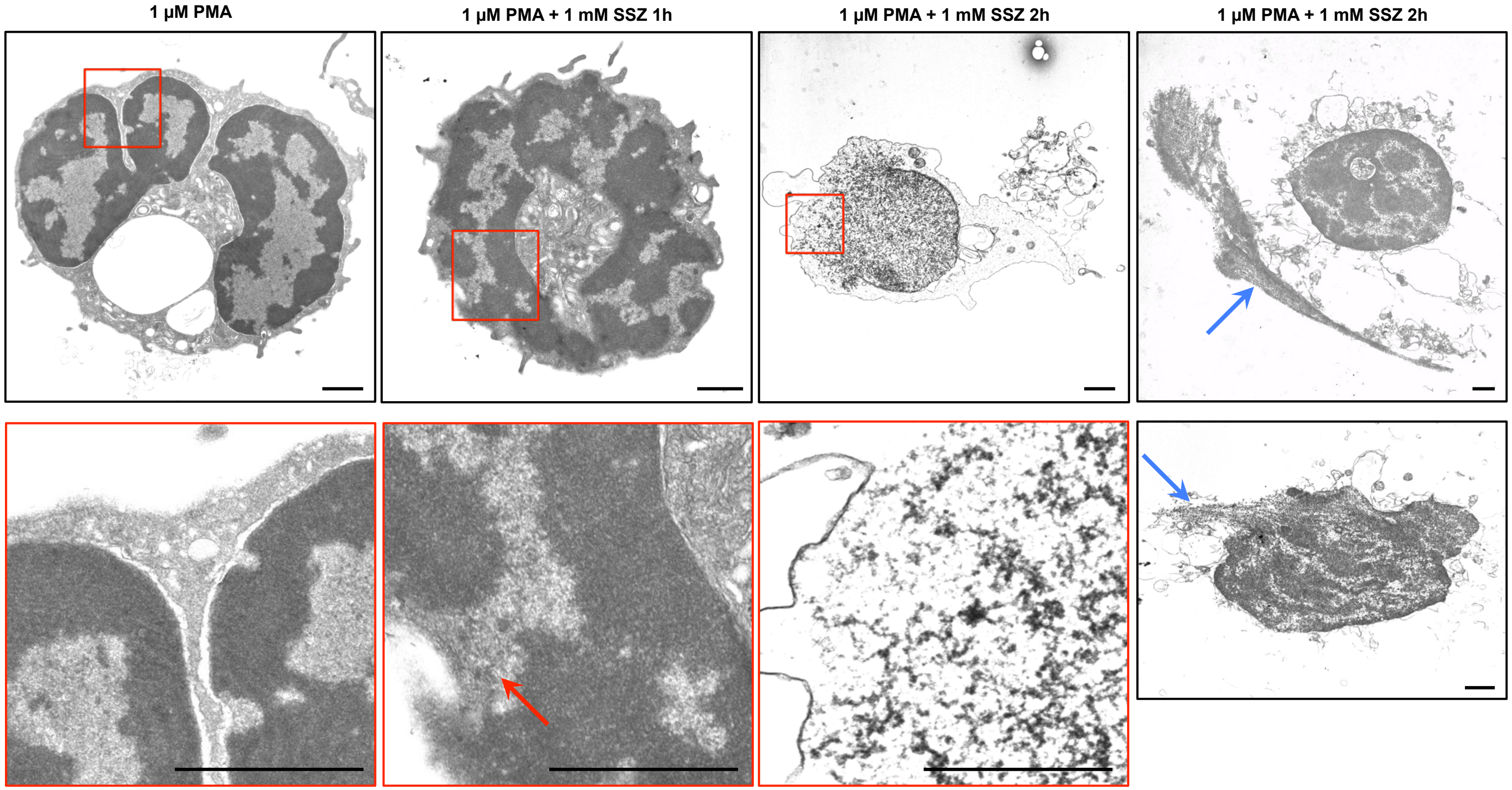


**Supplemental Figure. 3.** Transmission electron micrograph of PMA and SSZ-treated neutrophils. Mouse neutrophils were incubated with 1 μM PMA alone or 1 μM PMA+ 1 mM SSZ for indicated time. Cells were observed under transmission electron microscopy. Red arrow indicates loss of the nuclear envelope. Blue arrows indicate the prominent extracellular structures. Scale bars represent 1 μm. The data shown are representative images of triplicated samples.

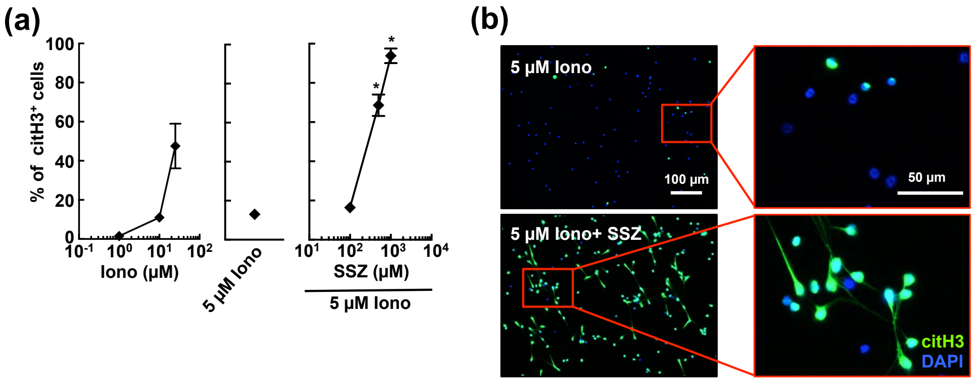


**Supplemental Figure. 4.** (a) Mouse neutrophils were stimulated with various concentrations of ionomycin alone (left) or 5 μM ionomycin + SSZ (right) for 4h. The proportion of cells undergoing NET formation was determined with anti-citH3 polyclonal antibody as described in (Figure 1h). Average values and the s.d. of triplicated samples in a single experiment are shown. *P< 0.01, one-way ANOVA, compared with 5 μM ionomycin. The data shown are representative of two independent experiments. (b) Mouse neutrophils were stimulated with 5 μM ionomycin alone or 5 μM ionomycin + SSZ for 4h. NET formation was visualized with anti-citH3 polyclonal antibody as described in (d). Original magnification, x20. The data shown are representative of two independent experiments.

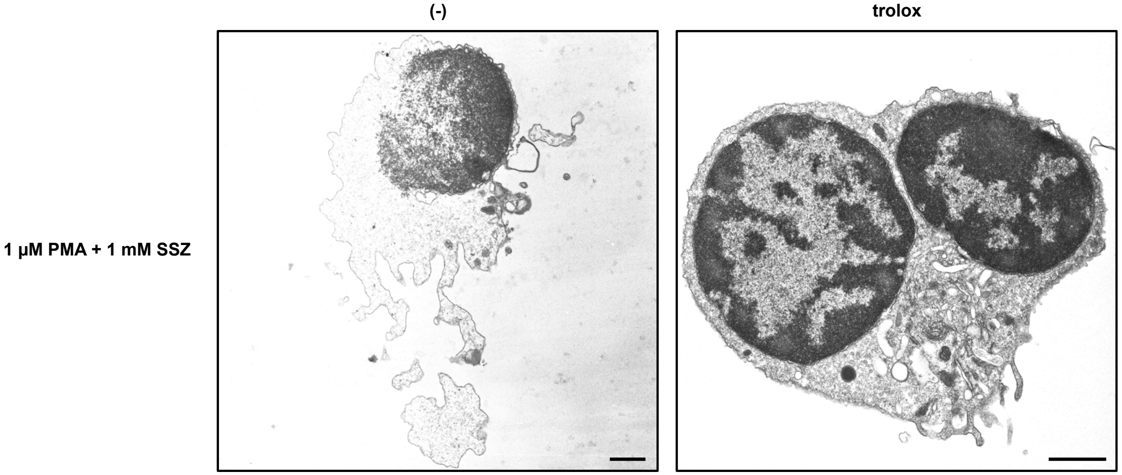


**Supplemental Figure. 5.** The effect of trolox on SSZ-induced NETosis. Mouse neutrophils were stimulated with 1 μM PMA + 1 mM SSZ in the presence or absence of 400 μM trolox. After 2 h, the cells were observed with transmission electron microscopy. Scale bars represent 1 μm. The data shown are representative images of triplicated samples.

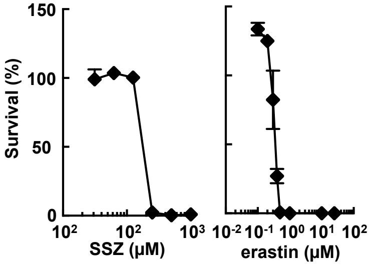


**Supplemental Figure. 6.** SSZ and erastin induce cell death in NIH3T3 cells. NIH3T3 cells were treated with various concentration of SSZ or erastin. Cell viability was assessed using Cell Counting kit-8 according to the manufacturer's protocols. Average values and the s.d. of triplicated samples in a single experiment are shown. The data shown are representative of three independent experiments.

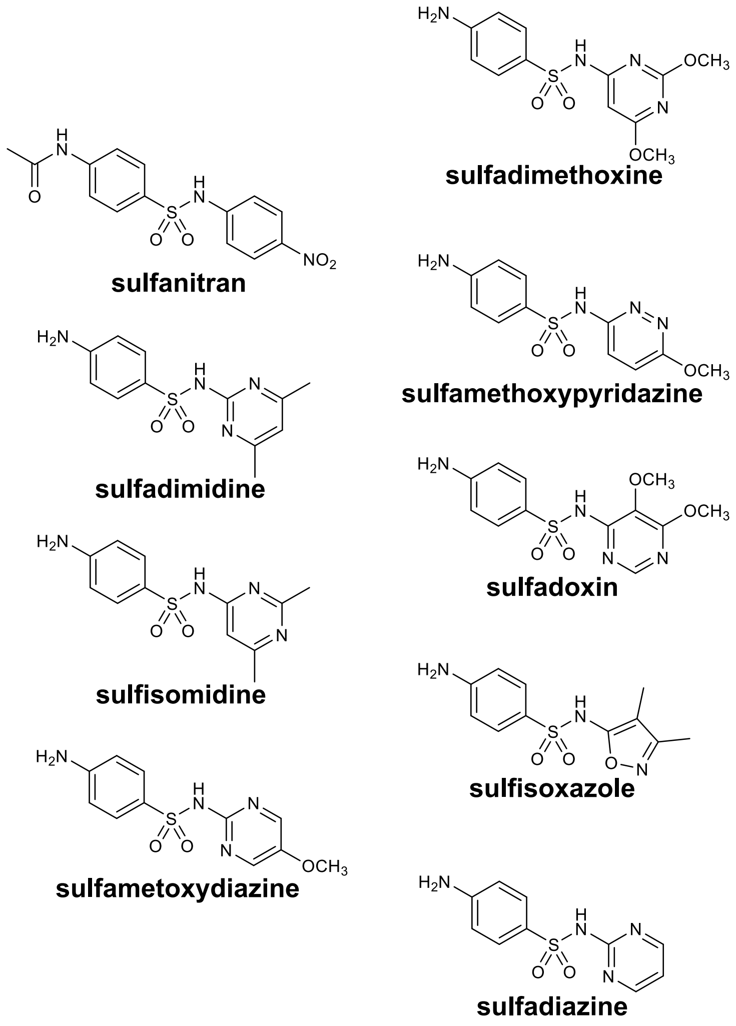


**Supplemental Figure. 7.** Structure of sulfa drugs having a variety of substituent on the sulfonamides

# Supplementary Information

## 1. General

NMR spectra were measured on a JEOL JNM-ECS 400 spectrometer. The proton chemical shift values are reported in parts per million and referenced to the proton resonance of $CD_3OD$ (3.31 ppm). The carbon chemical shift values are reported in parts per million and referenced to the carbon resonance of $CD_3OD$ (49.0 ppm). The data are presented in the following order: chemical shift, multiplicity (s = singlet, d = doublet, t = triplet, q = quartet, m = multiplet and/or multiple resonances, and br = broad), coupling constant, integration in natural numbers. ESI-MS was taken on Bruker microTOF-QII-RSL. IR spectra were recorded with Thermo Fisher SCIENTIFIC Nicolet iS5 spectrometer. Melting point was measured on a METTLER TOLEDO MP90 analyzer. Column chromatography was performed with silica gel 60N (40-50 μm) purchased from Kanto Chemical Co., Inc. Preparative thin-layer chromatography was performed with silica gel $60F_{254}$ purchased from Merck Co., Inc. Gel permeation chromatography was performed on YMC Forte HPLC system: column, YMC T2000.

## 2. Synthesis of Azo compounds NTP363 and NTP365

**(*E*)-2-hydroxy-5-((4-sulfamoylphenyl)diazenyl)benzoic acid (NTP363)**

4-Aminobenzenesulfonamide (103.9 mg, 0.603 mmol) was dissolved in aqueous 2 M HCl (1.81 mL) at ambient temperature. The solution was cooled at 0 °C. To this solution was added $NaNO_2$ (51.0 mg, 0.739 mmol) and the mixture was stirred for 15 min. To the diazonium salt mixture was added the solution of 2-hydroxybenzoic acid (85.6 mg, 0.619 mmol) in aqueous 2 M NaOH (3.0 mL). The resulting mixture was stirred at 0 °C for 30 min. The reaction mixture was quenched with saturated aqueous $NH_4Cl$ (1.0 mL), aqueous 2 M HCl (1.0 mL) and $H_2O$ 50 mL. The aqueous phase was extracted with diethyl ether (3 x 50 mL). The combined organic layers were dried over $MgSO_4$, and the solvent was removed *in vacuo*. The resulting residue was purified by flash column chromatography ($SiO_2$, eluent; toluene/MeOH = 3/1), preparative thin-layer chromatography ($SiO_2$, solvent system; toluene/MeOH = 3/1) and gel permeation chromatography (mobile phase; MeCN = 1, flow rate; 10 mL/min) to give desired product **NTP363** as an orange solid (15.4 mg, 0.0479 mmol, 8%).

$^1$H NMR (400 MHz, $CD_3OD$): δ 8.53 (d, $J$ = 2.4 Hz, 1 H), 8.04 (d, $J$ = 8.8 Hz, 2 H), 7.99-7.94 (m, 3 H), 6.95 (d, $J$ = 9.2 Hz, 1 H); $^{13}$C NMR (100 MHz, $CD_3OD$): δ 175.1, 167.3, 156.0, 146.1, 145.9, 129.2, 128.3 (2C), 127.5, 123.7 (2C), 120.5, 118.4; HRMS (ESI) calcd. for $C_{13}H_{10}N_3O_5S$ $[M-H]^-$ 320.0347, found 320.0349; ATR-FTIR ($cm^{-1}$): 3263, 2360, 2343, 1680, 1333, 1306, 1206, 1147, 911, 841, 804, 726, 668, 617; mp: 203 °C (decomposed), lit.[1] 220 °C (decomposed).

Ref. 1. Magidson, O. Yu.; Rubtsov, M. V. *Zhurnal Obshcheĭ Khimii* **1940**, *10*, 756-768.

**(*E*)-5-((4-((4-aminophenyl)sulfonyl)phenyl)diazenyl)-2-hydroxybenzoic acid (NTP365)**

4,4'-Diaminodiphenyl sulfone (152.0 mg, 0.612 mmol) was dissolved in aqueous 2 M HCl (1.84 mL) at ambient temperature. The solution was cooled at 0 °C. To this solution was added $NaNO_2$ (43.8 mg, 0.635 mmol) and the mixture was stirred for 15 min. To the diazonium salt mixture was added the solution of 2-hydroxybenzoic acid (85.1 mg, 0.616 mmol) in aqueous 2 M NaOH (3.06 mL). The resulting mixture was stirred at 0 °C for 30 min. The reaction mixture was quenched with saturated aqueous $NH_4Cl$ (1.0 mL), aqueous 2 M HCl (3.0 mL) and $H_2O$ 50 mL. The aqueous phase was extracted with diethyl ether (3 x 50 mL). The combined organic layers were dried over $MgSO_4$, and the solvent was removed *in vacuo*. The resulting residue was purified by Sep-Pak® tC18 (ODS, eluent; $H_2O$/MeOH = 9/1 to 1/5) and preparative thin-layer chromatography ($SiO_2$, solvent system; $CHCl_3$/MeOH = 3/1) to give desired product **NTP365** as an orange solid (4.3 mg, 0.0108 mmol, 2%).

$^1$H NMR (400 MHz, $CD_3OD$): δ 8.51 (d, *J* = 2.8 Hz, 1 H), 8.00 (d, *J* = 8.4 Hz, 2 H), 7.97-7.92 (m, 3 H), 7.64 (d, *J* = 9.0 Hz, 2 H), 6.93 (d, *J* = 8.8 Hz, 1 H), 6.70 (d, *J* = 9.0 Hz, 2 H); $^{13}$C NMR (100 MHz, $CD_3OD$): δ 175.0, 167.4, 156.3, 155.3, 146.1, 145.3, 130.9 (2C), 129.3, 129.1 (2C), 127.6, 127.4, 124.0 (2C), 120.5, 118.4, 114.6 (2C); HRMS (ESI) calcd. for $C_{19}H_{14}N_3O_5S$ $[M-H]^-$ 396.0660, found 396.0660; ATR-FTIR ($cm^{-1}$): 3375, 2360, 2341, 1628, 1593, 1488, 1458, 1432, 1295, 1143, 1104, 1072, 835, 806, 685, 668.

## 3. Proton and carbon NMR charts

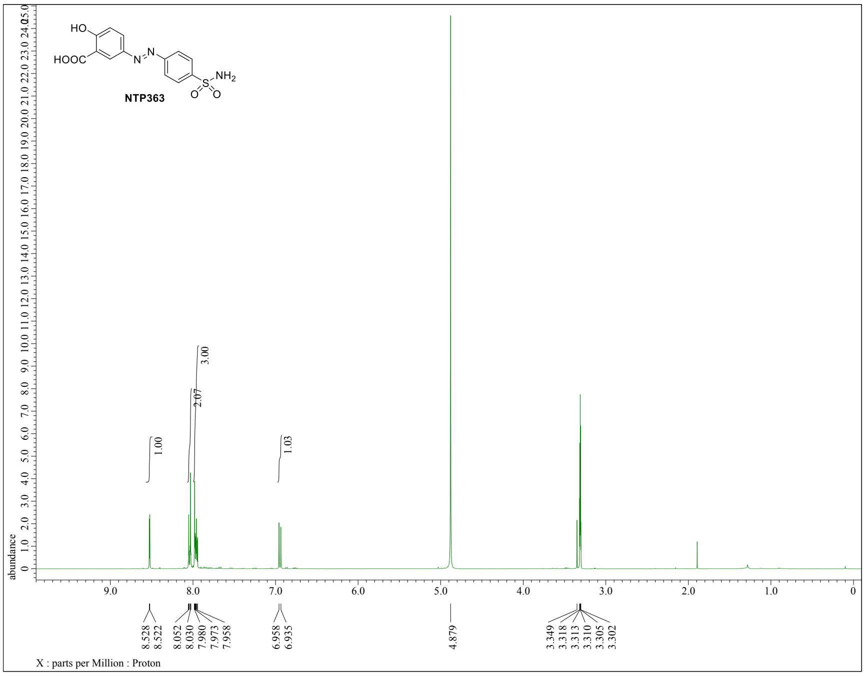


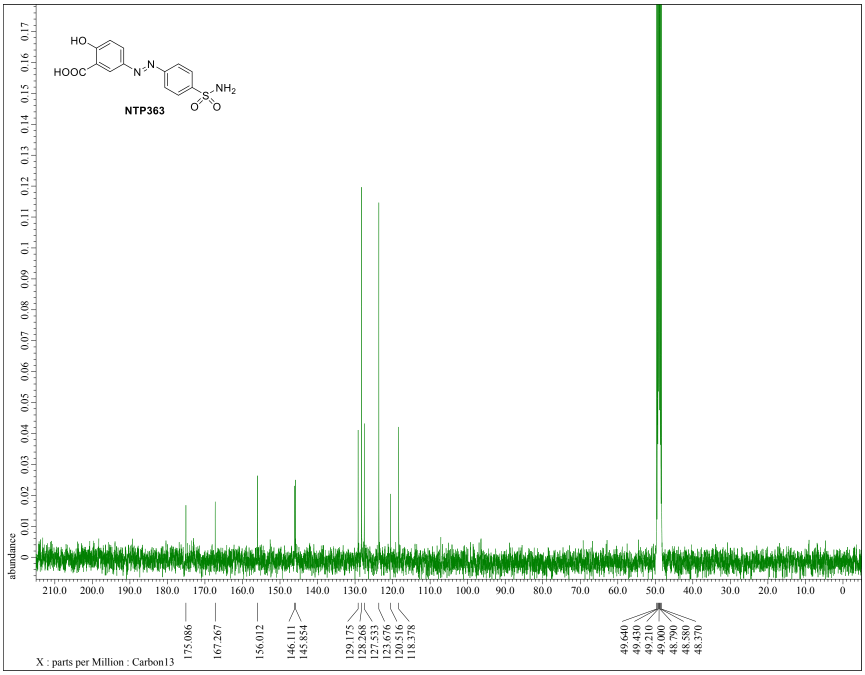

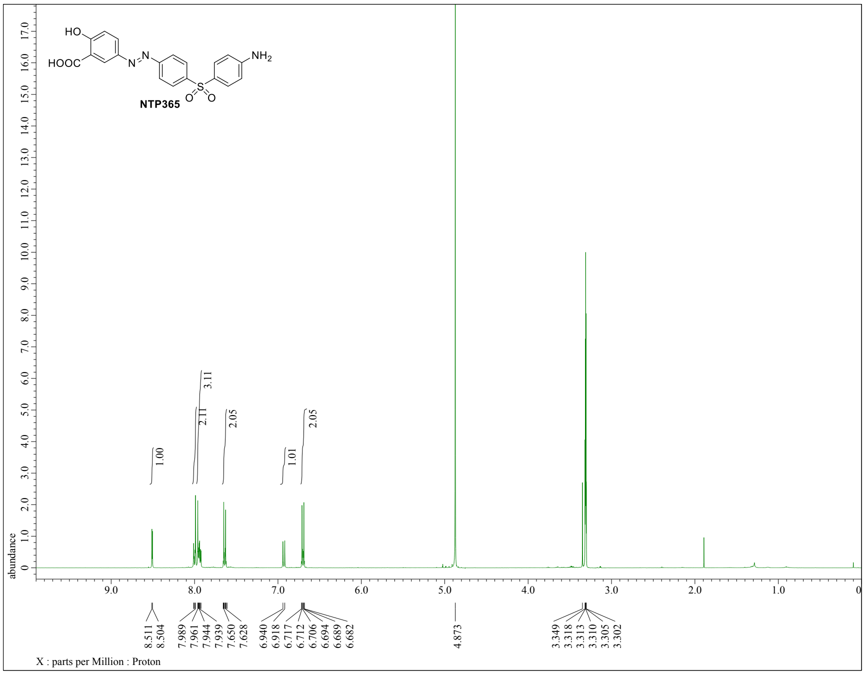
HO
HOOC
N
N
NH2
S
O
O
NTP365
abundance
X : parts per Million : Proton
1.00
2.11
3.11
2.05
1.01
2.05
8.511
8.504
7.989
7.961
7.944
7.939
7.650
7.628
6.940
6.918
6.717
6.712
6.706
6.694
6.689
6.682
4.873
3.349
3.318
3.313
3.310
3.305
3.302

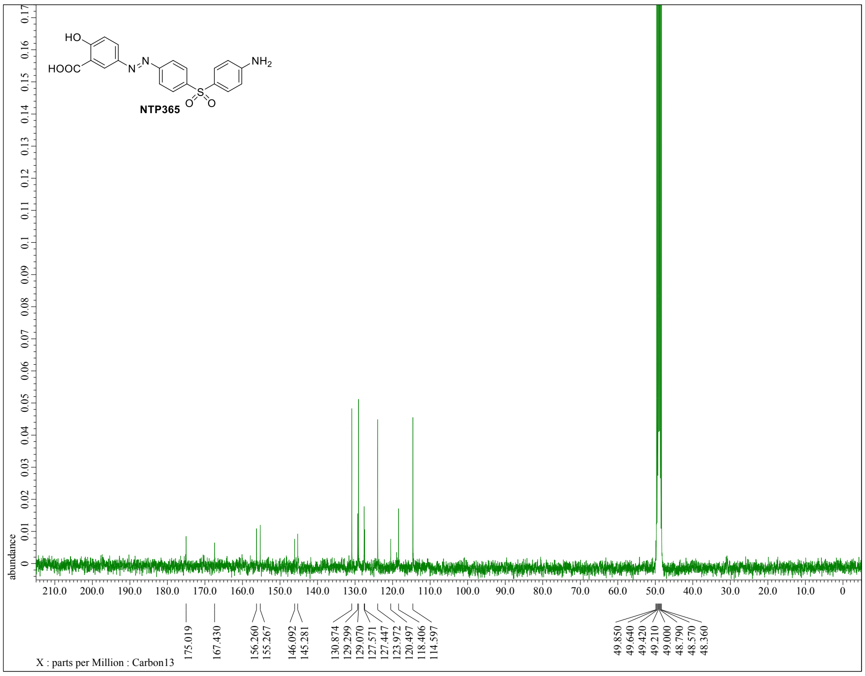
HO
HOOC
N
N
NH2
S
O
O
NTP365
abundance
X : parts per Million : Carbon13
175.019
167.430
156.260
155.267
146.092
145.281
130.874
129.299
129.070
127.571
127.447
123.972
120.497
118.406
114.597
49.850
49.640
49.420
49.210
49.000
48.790
48.570
48.360
